# Pharmacokinetics of magnetic iron oxide nanoparticles for medical applications

**DOI:** 10.1186/s12951-022-01510-w

**Published:** 2022-06-27

**Authors:** Julia Nowak-Jary, Beata Machnicka

**Affiliations:** Department of Biotechnology, Institute of Biological Sciences, University of Zielona Gora, Prof. Z. Szafrana 1, 65-516 Zielona Gora, Poland

**Keywords:** Iron oxide magnetic nanoparticles, Pharmacokinetics, Endocytosis, Blood half-life

## Abstract

Magnetic iron oxide nanoparticles (MNPs) have been under intense investigation for at least the last five decades as they show enormous potential for many biomedical applications, such as biomolecule separation, MRI imaging and hyperthermia. Moreover, a large area of research on these nanostructures is concerned with their use as carriers of drugs, nucleic acids, peptides and other biologically active compounds, often leading to the development of targeted therapies. The uniqueness of MNPs is due to their nanometric size and unique magnetic properties. In addition, iron ions, which, along with oxygen, are a part of the MNPs, belong to the trace elements in the body. Therefore, after digesting MNPs in lysosomes, iron ions are incorporated into the natural circulation of this element in the body, which reduces the risk of excessive storage of nanoparticles. Still, one of the key issues for the therapeutic applications of magnetic nanoparticles is their pharmacokinetics which is reflected in the circulation time of MNPs in the bloodstream. These characteristics depend on many factors, such as the size and charge of MNPs, the nature of the polymers and any molecules attached to their surface, and other. Since the pharmacokinetics depends on the resultant of the physicochemical properties of nanoparticles, research should be carried out individually for all the nanostructures designed. Almost every year there are new reports on the results of studies on the pharmacokinetics of specific magnetic nanoparticles, thus it is very important to follow the achievements on this matter. This paper reviews the latest findings in this field. The mechanism of action of the mononuclear phagocytic system and the half-lives of a wide range of nanostructures are presented. Moreover, factors affecting clearance such as hydrodynamic and core size, core morphology and coatings molecules, surface charge and technical aspects have been described.

**Graphical Abstract:**

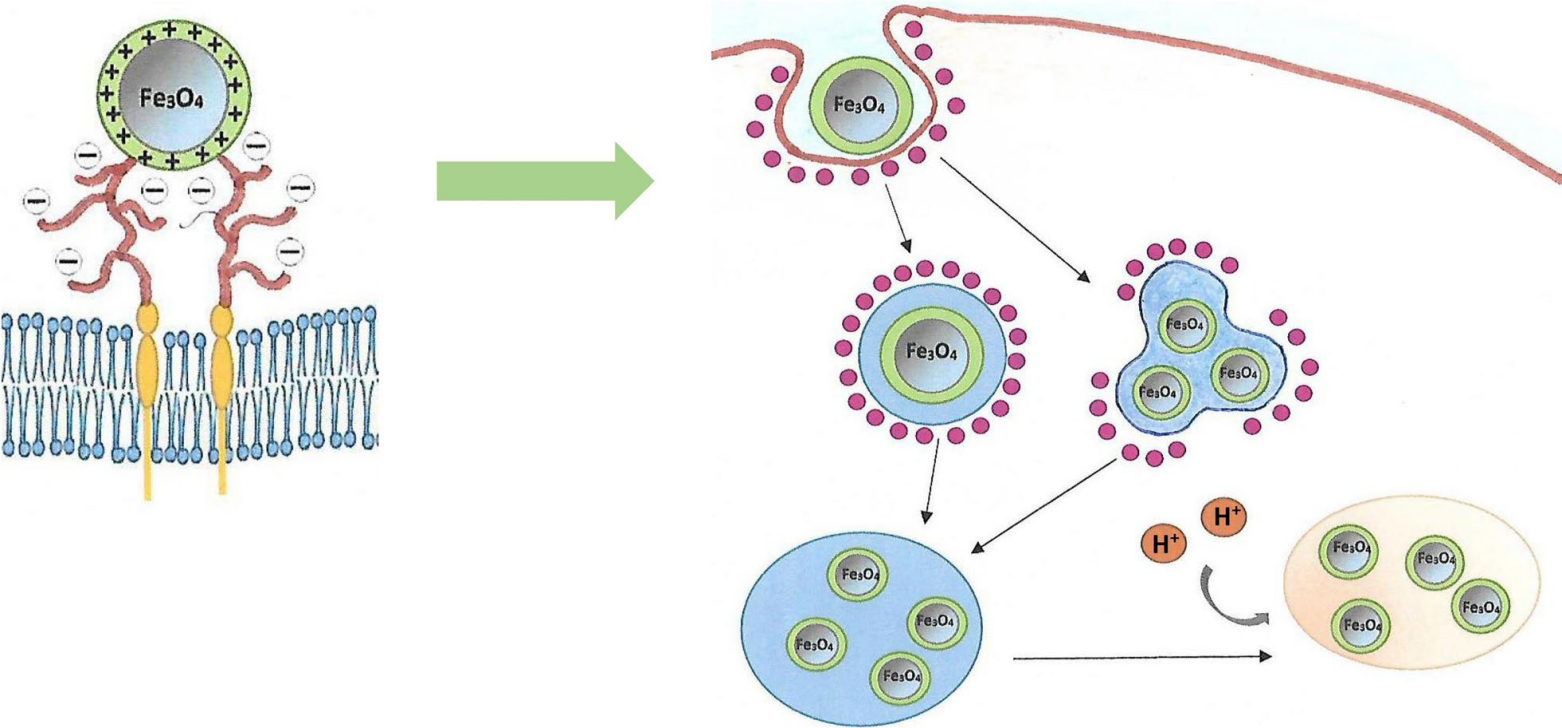

## Introduction

In the recent years magnetic iron oxide nanoparticles (MNPs) have been intensively developed and widely adopted for a range of biomedical applications such as tumors imaging (MRI) [1, 2], hyperthermia [3, 4], drug delivery [5, 6], gene therapy [7] and magnetic separation of cells or biomolecules [8, 9]. Irrespective of the specific applications, all magnetic nanostructures following in vivo administration are recognized by the host immunological mechanisms and eliminated from the body [10, 11]. Hence, there is always competition between clearance mechanisms and the long enough blood circulation time of MNPs sufficient to reach the particular organs and tissues. On the other hand, after any clinical diagnostic or therapeutic application, nanostructures should be easily metabolized and extracted from the organism [12]. Knowing the pharmacokinetics of the used magnetic nanostructures is crucial to enhancing their presumed functionality in any respective region of the body and minimizing their potential toxic effects due to undesirable biodistribution or accumulation.

Iron ions contained in magnetic nanoparticles are trace elements in the body. After digesting MNPs in lysosomes, iron ions can be incorporated into the natural circulation of this element. In the bloodstream, iron is bound by the transport glycoprotein called transferrin, and most of the absorbed iron is utilized by the bone marrow for erythropoiesis. On the other hand, ferritin is a cellular iron storage protein and a marker of iron charge in tissues [13].

The progress in the research on the use of magnetic nanoparticles for biomedical applications has shown that their pharmacokinetics and biodistribution are influenced by the size, shape, charge and, above all, surface chemistry of the nanostructures [14]. Therefore, depending on these factors, but also on the administration method, the expected pharmacokinetic behavior of MNPs may differ.

In this article, parameters playing the key role in the pharmacokinetics of functionalized MNPs are presented. It is suggested that all these parameters must be considered in order to develop magnetic nanostructures particularly useful in biomedical applications. The knowledge contained in this report is based on the research and reports findings in the field from the last two decades.

## Intravenously injected MNPs

Intravenous injection is the commonly used approach for administration of MNPs, especially for their use as MRI contrast agents and in the case of clinical oncology [15, 16]. The size of MNPs should be generally within the range from 1 to 100 nm and, in addition, the nanostructures must be coated with polymers, such as polyethylene glycol, dextran or silanes, to provide stability and avoid aggregation [17]. Dextran-coated iron oxide Fe_3_O_4_ nanoparticles sized 80–150 nm (Feridex®, US, and Endorem®, Britain) as well as dextran-coated MNPs sized 20–40 nm (Sinerem®, EU, Combidex®, US) are examples of clinically approved magnetic nanoparticles used for mononuclear phagocyte system imaging, lymph node and perfusion imaging as well as cellular labelling [18].

### Blood-half-life and mononuclear phagocytic system (MPS)

Blood or plasma half-life (*t*_*1/2*_) is the time needed for the concentration of magnetic nanoparticles in the blood or plasma to reach half of the initial concentration of the intravenous dose. The concentration of nanoparticles in the bloodstream decreases as a result of the elimination of MNPs through various organs such as the liver and the spleen. The particle size and coating type have a significant influence on the kinetics and blood half-life [19]. Due to magnetic properties of iron oxide nanoparticles, their half-life can be also defined as the time in which the MRI T_1_ (longitudinal) or T_2_ (transverse) relaxation times reduce to half their initial value [20]. The relaxation time of the magnetic nanoparticles characterizes the ability to retain the magnetization direction once the aligning field is removed. In other words, it is the time between the magnetic field removal and the protons returning to thermodynamic equilibrium. Some chosen values of functionalized MNPs half-lives in mammals are presented in Table 1.

**Table 1:** Blood half-lives (t_1/2_) of magnetic iron oxide nanoparticles (MNPs) coated with the different types of molecules following their intravenous injection into mammal models

Core size/hydrodynamic size (nm)	Name	Coating molecule	Model	Dose (mg Fe/kg)	*t*_1/2_	Applications/Investigation	Refs.
4-6/NA^1^	USPIO	Dextran	Rats	15	2 h	MRI of spinal cord	[233]
5/30	USPIO sinerem	Dextran	Rats	11.2	4 h 30 min	Tumor MRI	[234]
NA/15	Fractionated Feridex	Dextran	Rabbits	4.8	15.9 h	MR imaging for atherosclerosis	[235]
NA/50-80	Amino-dextran SPIO-micromod	Dextran 20 kDa	Mice	4	5-60 min	Protein absorption analysis	[236]
3-5/60-80	Resovist (SHU 555 C, ferucarbotran)	Carboxydextran	Rats	5.6	56 ± 17 min	Imaging of inflammatory bowel disease	[237]
30/30-70	Nanoworms	Dextran-PEG^2^	Mice	3	16–19 h	Tumor targeting	[67]
7/NA	NA	Chitosan-chlorotoxin-cy5.5	Mice	6.67^3^	7–8 h	Cancer targeting and imaging/NIR fluorescence scanner for half-blood life	[24]
70/NA	NA	Silica + PEG	Rats	5.7	2.5 h	General MRI	[20]
12/NA and 15/NA	MF66 and OD15	DMSA^4^	Pigs	0.5 -2	15 min	Breast and pancreatic cancer	[238]
NA/46 and 53	PDS1 and PDS8	Dextran-PEG	Mice	100	< 1 h	NA	[239]
NA/29	NA	EDT^5^	Mice	5	6 min	Brain targeting	[232]
NA/65	NA	Dextran	Mice	2	150 min	Tumor targeting	[224]
5-10/194	SPIO-alginate	Alginate	Rats	6.1212.23	0.25 h0.59 h	MR liver imaging	[134]
5/15-50	Ferumoxtran-10 (USPIO, AMI-227)	Dextran	Humans	2.6	> 24 h	MRI for detection in lymph nodes	[50]
5/62-80	Ferumoxides (SSPIO, AMI-25, SHU 555 A)	Dextran/Carboxydextran	Humans	1.16 – 11.6	3.9 – 8 min	MRI for metastatic lesion detection in liver	[48]
10/NA35/NA	LUSPIOLSPIO	PEGgylated lipid	Mice	3.9	1.41 h1.01 h	Imaging of oxidation-specific epitopes within the arterial wall	[40]
5.6/12	NC100150	Oxidized starch	Humans	1, 2 and 5	2-3 h^6^	Positive-contrastMR angiography	[227]
7/74.9	PC SPION	Oleic acid/Encapsulation into phosphatidylo-cholinemicelles	Rats	0.15	10 h	MRI contrast agents/drug delivery	[226]
26/78	LS-008	PMAO^7^-PEG	Rats	5	4.2 h	MPI tracer	[170]
3.2/116.2	NA	PEG-cysteine	Rats	NA	6.2 h	T_1_-weighted MR imaging	[88]
NA/34.1-35.9	IONP-ICG	Dextran-ICG^8^-PEG	Mice	55.8	164-197 min	Imaging of macrophages in atherosclerotic plaques	[228]
NA/50	MNP-VEGF^9^@Dox^10^	Albumin-PEG-VEGF@Dox	Rats	5	14.6 h	Targeted theranostics of breast cancer	[89]
9/16	BFNPs	Fluorescent carbon	Mice	3.33^3^	1.36 h	Photothermal therapy for tumor treatment	[229]
8-12/30	RGD10-NGR9-USPIO	Dextran-RGD10-NGR9-peptides	Mice	25	6.2 h	MRI of tumor angiogenesis	[230]
30-35/94	MNP@PES-Cy7/2-DG	Poly(4-styrenesulfona-te)-Cyanine7/2-deoxyglucose-polyethylene glycol	Mice	NA (0.075 mg NPs per mouse)	1.61–2.07 h^11^16.2–24.56 h^12^	Trimodality imaging-guided intracellular photo-magnetic hyperthermia therapy	[240]
5/140-230^13^	Raspberry SPIONs	Oleic acid/GCPQ^14^	Mice	32.5	28.3 min	MRI contrast agents	[97]
14/40	IONP@PMSEA^15^	Oleic acid/PMSEA	Rats	10	5.15 h	Potential delivery agents for therapeutics and diagnostics	[94]
11/22	Fe_3_O_4_-PEG-5Ab^16^	PEG-5Ab	Mice	10	6.96 h	Targeted imaging and enhanced treatment of NHL^17^	[241]
NA/64	MCP^18^-PEG10K	PEG 10 kDa	Rats	2.795.59	1.8 min5.2 min	MPI tracers	[80]
NA/84.1	MCP-PEG10K2	PEG 10 kDa (double layer)	Rats	2.79	62.1 min	MPI tracers	[80]
20.7-22.6/54-76	RL-1	PEG-silane	Mice	6.67^3^	6.99 h	MPI tracers	[231]
10/154	PEG-starch-IONPS	PEG-starch	Mice	12	2.7 h	Photothermal therapy (PTT) agents	[84]
13/178	PTX^19^@FA^20^@PEG/PEI^21^-SPIONs	PTX-FA-PEG/PEI	Rats	NA	3.41 h	PTX delivery system	[85]

^1^NA – not available; ^2^PEG- polyethylene glycol; ^3^assuming that each mouse weighed 30 g; ^4^– dimercaptosuccinid acid, ^5^– ethylenediaminetriacetate, ^6^- depending on the dose, ^7^- poly(maleic anhydride-alt-1-octadecene), ^8^- indocyanine green, ^9^- vascular endothelial growth factor, ^10^- doxorubicin, ^11^- distribution phase, ^12^- elimination phase, ^13^- 5 nm SPIONs clustered into larger raspberry shape, ^14^- N-palmitoyl-N-monomethyl-N,N-dimethyl-N,N,N-trimethyl-6-O-glycolchitosan, ^15^- poly[2-(methylsulfinyl)ethyl acrylate], ^16^- RTX(rituximab)antibodies, ^17^- non-Hodgkin lymphoma, ^18^- magnetic multicore particles, ^19^- paclitaxel, ^20^- folic acid, ^21^- poly(ethyleneimine)

When administered intravenously, MNPs are selectively engulfed by the cells which are a part of the mononuclear phagocyte system (MPS), also called the macrophage or reticuloendothelial system (RES) [21]. However, it should be noted, that although the nomenclatures MPS and RES are commonly used interchangeably in relation to macrophages, there are reports showing that the system described as RES in the liver involves sinusoidal endothelial cells (LSEC), but not liver macrophages (Kupffer cells) [22].

**Fig. 1:**
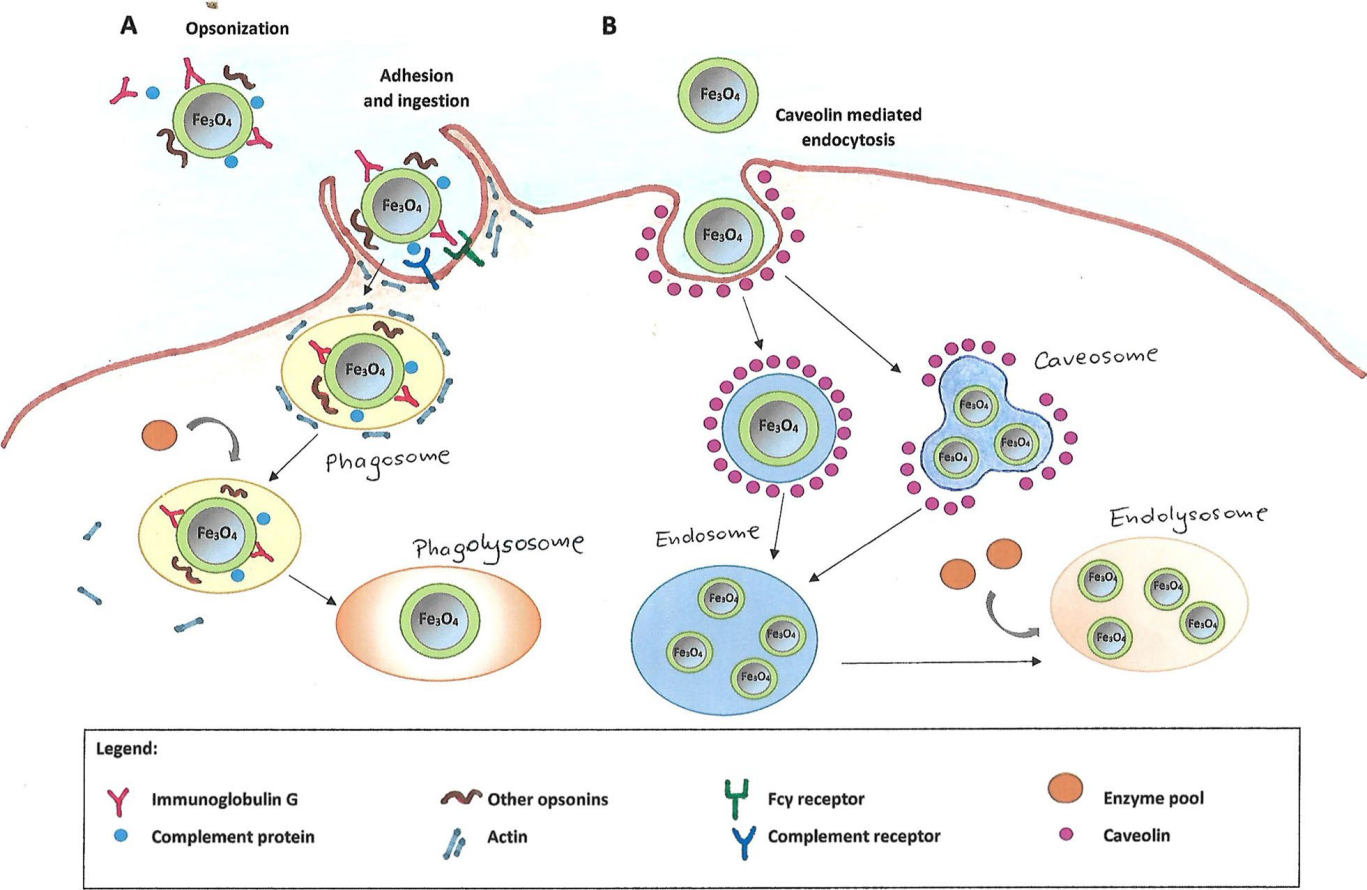
Magnetic nanoparticle internalization by opsonization and phagocytosis (**A**) and caveolin mediated endocytosis (CVME) (**B**)

MPS cells arise from the precursors present in the bone marrow. These precursors develop into i.a. phagocytic cells called monocytes which then circulate in the blood. Some monocytes remain in the main bloodstream, but most of them penetrate specific body tissues, where they develop into larger phagocytic cells known as macrophages [23]. Most of macrophages remain as stationary cells within tissue, where they filter out and destroy foreign particles. MPS cells differ in term of their occurrence and names; for example the so called dendritic cells are found in many tissues, including the lungs, the skin, and the gastrointestinal tract, whereas Kupffer cells are localized in the liver [21, 22]. Distinct macrophages also exist in secondary lymphoid organs, including the spleen and lymph nodes [23]. All these MPS cells clear the body of pathogens, such as bacteria, viruses, old and abnormal cells, as well as foreign bodies, for example injected nanoparticles. The main organs involved in the MNPs clearance are the liver and the spleen [24], however, in the case of the administration of high doses of nanoparticles, the presence of the excess MNPs was also found in other tissues such as lungs and adipose tissue [25]. The uptake of MNPs by the macrophages is usually preceded by opsonization (Fig. 1A). The process involves the attachment of specific proteins to the surface of the nanostructures. Opsonization takes place in the bloodstream immediately after the injection of the nanoparticles. The most abundant opsonins are immunoglobulins (Ig G and M), complement components (C3, C4, C5) [26] and blood serum proteins (such as albumins, fibrinogen, fibronectin, C-reactive protein, type-I collagen) [27]. Due to this specific labeling, MNPs become visible to macrophages and attach to their surface through specific receptor-ligand interactions, which results in the formation of a recess. Subsequently, the created phagosome carries the nanoparticle through the cytoplasm and, following actin depolymerization, it becomes accessible to lysosomes [28]. Ultimately, after fusing with lysosomes, it forms a phagolysosome containing many enzymes in acidic environment. The second main uptake pathway for magnetic nanoparticles, apart from phagocytosis, is the process of pinocytosis, for which the major intermediary protein is caveolin [29] (Fig. 1B). The interactions are the signals to initiate the cascade mediated by GTPases, which triggers off actin assembly, forming a cavity on the macrophage surface that encloses over the nanoparticle, effectively engulfing it.

**Fig. 2:**
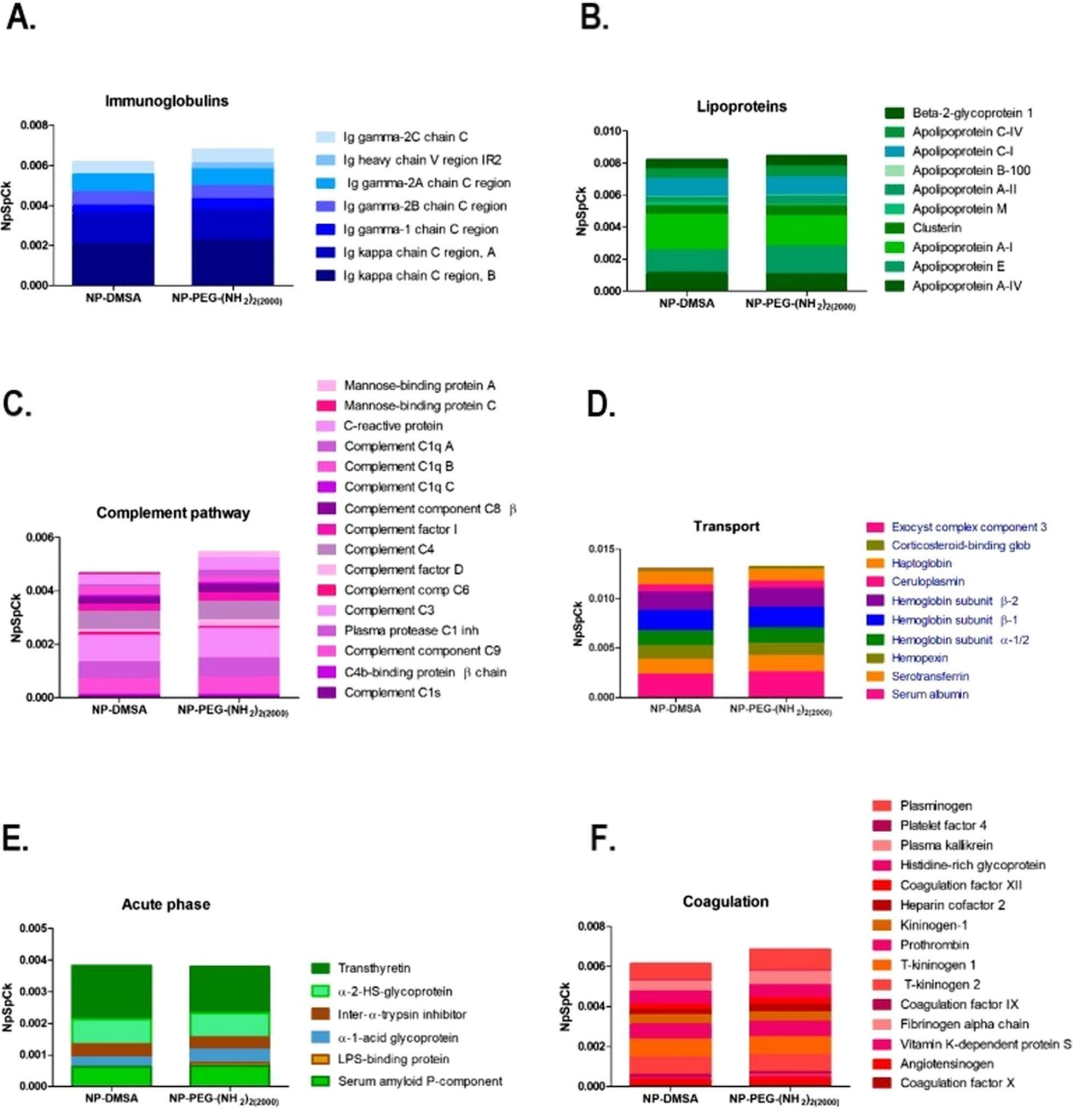
Semiquantitative analysis of the protein corona composition of NP-DMSA and NP-PEG-(NH_2_)_2(2000)_. Inmunoglobulins (**A**), Lipoproteins (**B**), Complement pathway (**C**), Transport (**D**), Acute phase (**E**), Coagulation (**F**). Republished from Ref. 31 under the terms of the Creative Commons Attribution Licence (CC BY) (http://creativecommons.org/licences/by/4.0/)

Although a wide range of sizes and materials have been used to prolong the circulation time or to uprate target specificity of nanoparticles, their applications are still limited by MPS/RES [30]. Undoubtedly, in order to rationally design nanoparticles for medical applications, it is necessary to understand the mechanism of formation of the protein corona and its composition. Ruiz et al. [31] investigated, by means of proteomic analysis, the formation and composition of the protein corona around magnetic nanoparticles coated in two ways: in the first case using dimercaptosuccinic acid (DMSA), and in the other – by means of a diamine (PEG)-derived molecule (2000 Da) which is widely used for providing a long circulation time [32, 33]. Semiquantitative analysis of the protein corona composition of the above-mentioned nanoparticles is shown in Fig. 2 [31]. Kinetic studies have shown that the corona formation around the MNPs accomplished in two main stages. Firstly, after the nanoparticles have been introduced into the biological environment, the initial corona is formed by the biomolecules which are the first to encounter the MNPs. Next, in the second stage, the corona composition is changing dynamically because of the competition among proteins. Thus protein–protein interactions may have a significant effect on the course of the process. It is also widely known that the structure and composition of the protein corona changes constantly [34]. Quantitatively dominant proteins bind first, but they are eventually displaced by those with higher affinity. Cedervall et al. [35] reported that albumin and fibrinogen exhibited higher rates of both association and dissociation than many other plasma proteins, including apolipoprotein A-I. Consequently, albumin and fibrinogen might dominate on the MNPs surface initially and for a short time, but subsequently they would be replaced by the proteins with higher affinity and slower kinetics, for example apolipoprotein A-I. Only when these proteins with higher affinity are not enough to cover the surface of all nanoparticles, lower affinity proteins, such as albumin, can also be found in the protein corona. As a result, the composition of the protein corona is very difficult to ascertain, still its composition may be studied by the techniques such as Mass Spectrometry and Electrophoresis SDS-PAGE. Since this is a key factor for RES/MPS recognition of nanoparticles, further studies are required for a more complete understanding of the in vivo behaviour of MNPs.

### The factors influencing the pharmacokinetics of iron oxide nanoparticles

#### Size and shape

Hydrodynamic size (d_H_) is one of the primary factors determining the pharmacokinetics of nanoparticles [19, 36, 37]. D_H_ is the size including any solvent molecules attached to the surface of the nanoparticle and it is traditionally measured using DLS (Dynamic Light Scattering) technique [38]. Generally, it has been proved that nanoparticles with the hydrodynamic sizes within the 15–100 nm range are optimal as MNPs of these sizes show the longest circulation time in the bloodstream and thereby have a greater chance of reaching other organs and targets, such as, for example, the brain, arterial walls, lymph nodes or tumors [39–42]. Larger particles (d_H_ > 100 nm) are readily picked up by the phagocytic cells and accumulated in the liver and the spleen [40, 43], whereby particles of the > 200 nm size diameter show higher uptake rates by the spleen in comparison to the liver [44, 45]. Very small nanoparticles (< 10–15 nm) are eliminated by the kidneys [46, 47]. Generally, ultrasmall MNPs nanoparticles enter the blood vessels of the glomeruli in the nephrons and are eventually extracted in urine by the ureter and then by the urinary bladder. However, it should be noted that even if the size of nanoparticles determined by DLS is within the 10–100 nm range, the sample may also contain larger aggregates. This may be indicated by the high polydispersity index (PDI) which is determined during measurements using the DLS technique. This indicator is usually within 0.1–0.7 range. Low values of the index mean a narrow range of the size distribution of MNPs, and thus a homogeneous sample. However, the higher the PDI values, the more likely it is that the sample is not uniform and contains larger aggregates of particles.

An example of the nanoparticles in the case of which the dependence of the half-life and their size is clearly apparent are Ferumoxides [48, 49] and Ferumoxtran-10 [50] – MRI agents. Both have similar dextran-coating, but Ferumoxtran-10 composed of smaller nanoparticles (d_H_ = 15–50 nm) is characterized by a much longer circulation time (human blood half-life between 24 and 36 h) than Ferumoxides with d_H_ of 62–80 nm and human blood half between 3.9 and 8 min. Consequently, Ferumoxtran-10 nanoparticles have easier access to lymph nodes, the brain and osteoarticular tissues, whereas Ferumoxides - with larger nanoparticles - is rapidly cleared from the bloodstream as a consequence of its uptake by the Kuppfer cells in the liver [48].

There are many reports regarding the influence of the hydrodynamic nanoparticle size on their clearance. For example, the circulation times of nanoparticles coated with glucuronic acid and sized 50, 100 and 250 nm were studied. [51]. The results revealed the following tendency: the smaller MNPs, the longer circulation time, however the difference between the half-life of 50 and 100 nm particles was significantly larger than between 100 and 250 nm ones. These results are consistent with previous studies [52]. Briefly, non-stealth cyanoacrylate particles of 85, 172 and 242 nm in size showed nearly the same average half-life in the blood. Also abundantly PEG coated nanoparticles sized 80 and 200 nm showed similar pharmacokinetics [53]. According to Zelepukin et al. [51], in some cases this phenomenon may be accounted for by their extremely rapid clearance. On the other hand, it may be caused by the increase in the amount of the particles under the same weight doses and by the different particle uptake mechanism [54] e.g., macropinocytosis and phagocytosis attenuation and the increased role of clathrin-mediated and other types of endocytosis when MNPs size is decreased. However, He et al. [53] explained that the impact of nanoparticle size on the blood circulation time is significant, but only when PEGylation is ineffective, e.g. with less density. On the other hand, if the efficiency of the PEG coating of nanoparticles is high, their size may have a much smaller effect on the MNPs half-life.

Other researchers have also noted that the size of nanoparticles and their circulation time in the blood are not always correlated in a simple, unambiguous way. More often it is the resultant of the appropriate size, charge, and density of the “shell”. For example magnetic nanoparticles varied in size (10, 20 and 31 nm) and coated with PEG terminated by bisphosphonate anchoring groups (neridronate) were tested [55]. The Fe_3_O_4_@PEG-Ner-10, -20, and -31 particles were removed from the bloodstream within 5, 14, and 4 h, respectively, and no nanoparticles were detected in the blood at 25 h post-injection. Hence, there was no visible relationship between the size of the nanoparticles and their circulation time. The authors explained that the reason for the shorter half-time of nanoparticles with a smaller hydrodynamic diameter (Fe_3_O_4_@PEG-Ner-10) was probably due to their lower negative zeta potential, which resulted in their aggregation, and thus an increase in the degree of opsonization and elimination. On the other hand, nanoparticles with a larger hydrodynamic diameter (Fe_3_O_4_@PEG-Ner-31) had a lower PEG density on their surface than nanostructures with a diameter of 20 nm, leading to lower environmental stability. Thus, the long circulation time of Fe_3_O_4_@PEG-Ner-20 nanoparticles was probably caused by the combination of PEG-neridronate coating and the proper size, coating density and the charge of the particles. Moreover, the protein corona formed on nanoparticles in the bloodstream can strongly affect their behaviour in biological systems, in particular the interaction with blood cells, thereby consequently affecting the removal of the particles from the blood [56]. The influence of factors other than size, such as surface chemistry and charge on circulation time, is described in the following subsections.

Theoretically, larger crystalline iron oxide core sizes (d_C_) should lead to larger hydrodynamic sizes. However, it should be borne in mind that there are large magnetostatic and dipolar interactions among naked iron oxide nanoparticles, which results in their aggregation. The effect of MNPs core size on their circulation time was investigated by Briley-Saebo et al. [40]. Developing the method of atherosclerotic lesions imaging using magnetic iron oxide nanoparticles coated with PEGylated lipids, they demonstrated that MNPs with the iron core size of 10 nm exhibited longer half-life in the mouse bloodstream (t_1/2_ = 1.41 h) compared to analogous nanoparticles, but with a larger core diameter (d = 35 nm, t_1/2_ = 1.01 h).

**Fig. 3:**
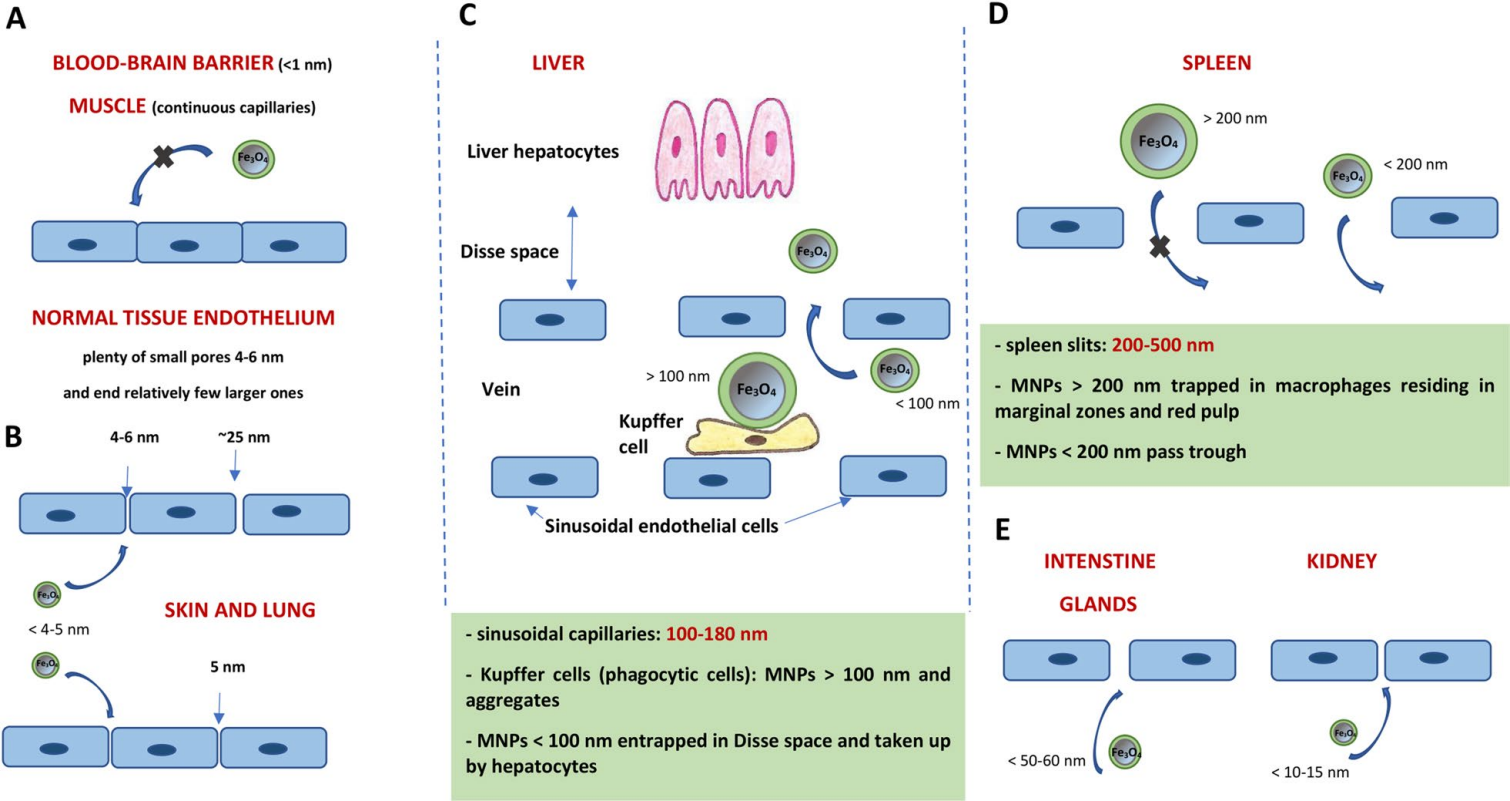
Delineation showing the size dependent physiological barriers against magnetic nanoparticles blood circulation. Even the smallest magnetic nanoparticles do not cross the blood-brain barrier and the blood vessel epithelium in the muscles, as long as the tissues are not cancerous (**A**). Nanoparticles with a diameter of about 5 nm or less are able to penetrate through small pores such as in the epithelium of the lungs and skin (**B**). Sinusoidal capillaries in the liver are fenestrated (100–180 nm) and lined with the Kupffer cells which quickly uptake large nanoparticles (> 100 nm) or agglomerates tagged with opsonins, whereas smaller nanoparticles (< 100 nm) are captured and hidden in the Disse space from where they can be collected by hepatocytes (**C**). Nanoparticles larger than about 200 nm get trapped in the marginal zones and the red pulp of the spleen, where they are absorbed by splenic macrophages (**D**). In the kidneys, nanoparticles with d_H_ < 10–15 nm in diameter are filtered out, whereas nanoparticles with d_H_ < 50–60 nm can penetrate through the pores in the intestines and glands (**E**)

Taking into account the medical properties of nanoparticles, it was reported that MNPs of approximately 12 nm were optimal for cancer therapy due their excellent tumor penetration [57], whereas nanomedicines of 50 nm were suitable for overall tumor tissue accumulation and retention [58]. Moreover, MNPs removal from the bloodstream in the human body depends largely on the pores sizes in the epithelium of blood vessels, which are as follows [5]: normal tissue endothelium (plenty of small pores of approx. 4.5 nm and relatively few larger ones of approx. 25 nm), tight-junction capillaries (for example blood–brain barrier *<* 1 nm), continuous capillaries (for example muscle, skin and lung: approx. 5 nm), fenestrated capillaries (kidneys, intestines, glands: 10–60 nm) and sinusoidal capillaries (liver, spleen, bone marrow: 100 nm – 1 μm). The maximum size of nanoparticles that will allow them to penetrate cell membranes is 1 μm. Schematic representation of the nanoparticle size-dependent barriers is shown in Fig. 3.

The findings regarding the impact of the MNPs size on their pharmacokinetics described above, concern spherically shaped nanoparticles. However, many studies indicate that the particle shape is as important as size, or even more so [59–63]. It was shown that for each type of nanostructure, a large length-to-width ratio translates into longer circulation time for nanoparticles [64]. The phenomenon is caused by the lesser uptake by macrophages due to an opsonin-independent phagocytosis [65]. This principle also applies to magnetic iron oxide nanoparticles [66]. For example, specific iron oxide called “nanoworms” showed prolonged circulation time up to 19 h [67]. There are also reports showing that oblate spheroid nanoparticles exhibit longer circulation time than spherical nanoparticles of the same volume [68]. In general, nanoparticle internalization, in the process of phagocytosis, for example, is a complex of three shape– and size–dependent parameters: (i) particle surface-to-cell membrane contact area, (ii) strain energy for membrane deformation, and (iii) sedimentation or local particle concentration at the cell membrane [69]. However, in order to understand the effect of the proportions of one-dimensional nanoparticles on the clearance mechanism, and thus increase their half-life and improve pharmacokinetics, meticulous comparative analyses are needed [70].

**Fig. 4:**
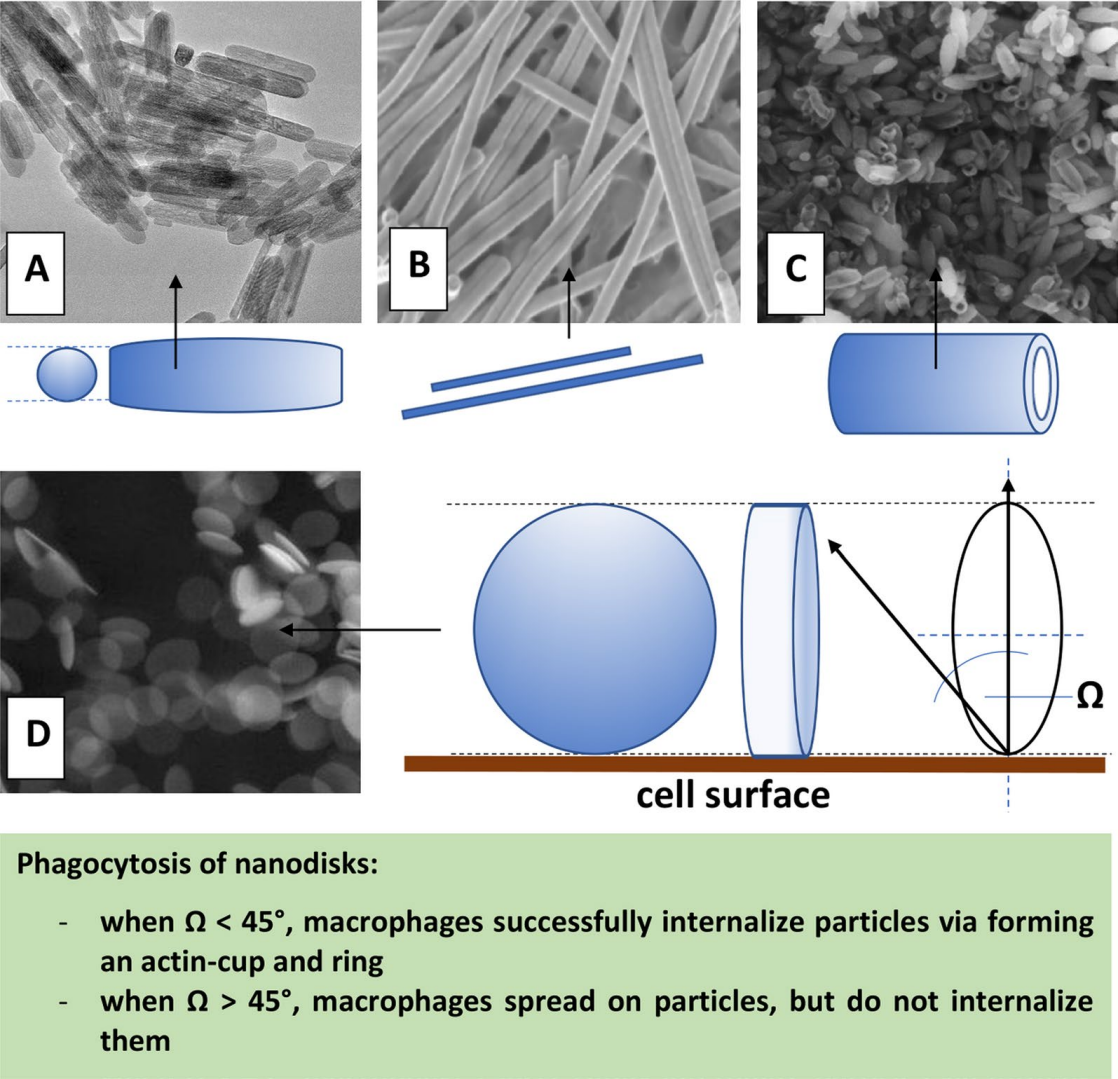
The main non-spherical shapes of MNPs: nanorods (A), nanowires (B), nanotubes (C), nanodisks (D). SEM images A, B, D republished from Ref. 242, 243, 244, respectively, under the terms of the Creative Commons Attribution Licence (CC BY) (http://creativecommons.org/licences/by/4.0/); SEM image C republished from Ref. 245 with permission of Elsevier

The main types of shape of magnetic nanoparticles used in biomedical applications are shown in Fig. 4.

#### Coating molecules

Since the un-coated magnetic nanoparticles are colloidally unstable and are quickly eliminated from the bloodstream by the MPS system following aggregation, molecules coating the MNPs surface play a crucial role in improving their pharmacokinetic properties. Polymer shells prevent protein binding by reducing interactions, and thereby prolonging the circulation time of the MNPs in the bloodstream [71].

##### Polyethylene glycol (PEG) and derivatives

**Fig. 5:**
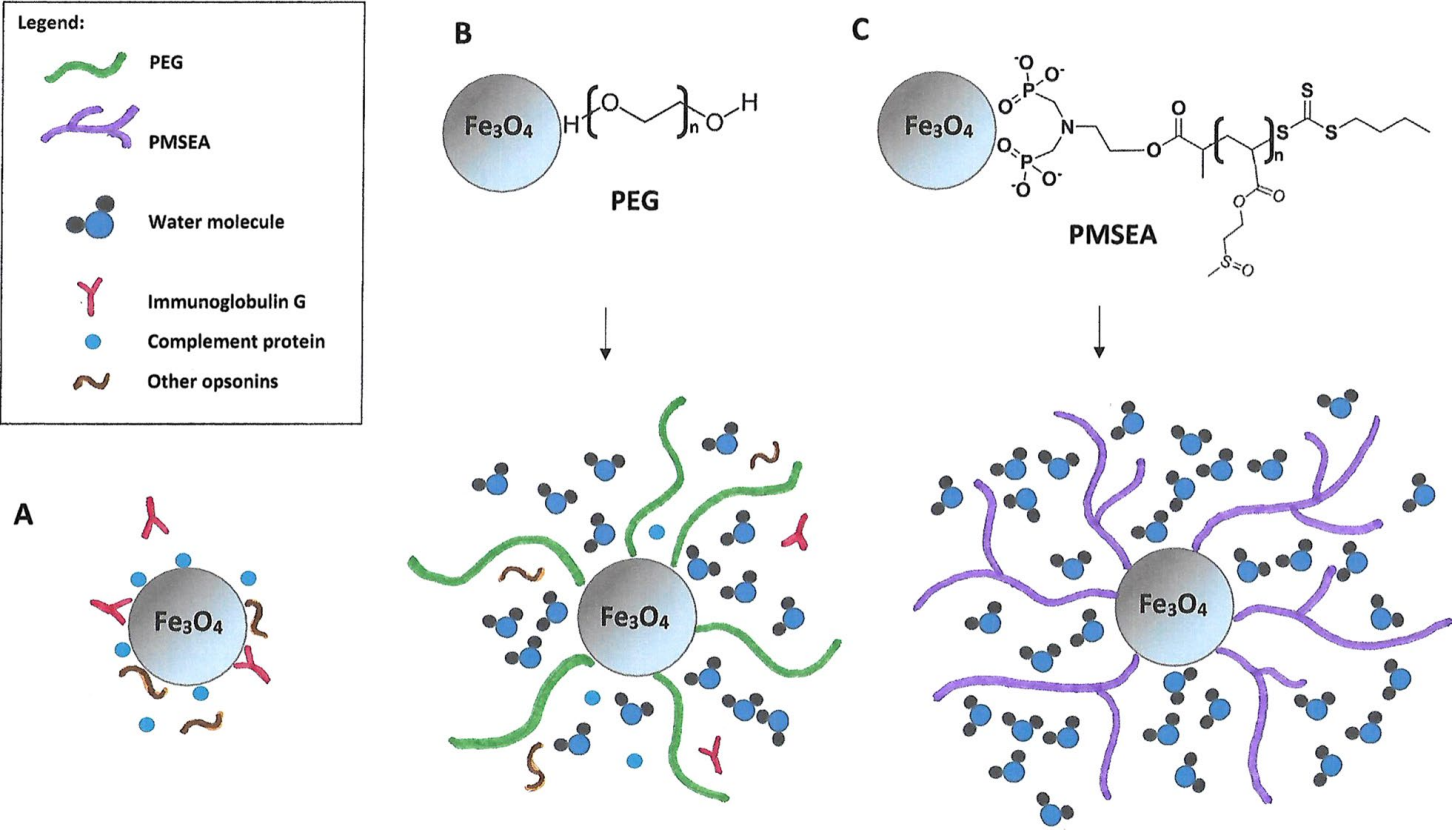
The uptake of the MNPs by the macrophages is usually preceded by opsonization, which involves the attachment of specific proteins on the surface of the nanostructures (**A**). The „stealth” effect of the one of the most popular coating materials: PEG [poly(ethylene glycol) is explained by the high level of hydratation of the hydrophilic polyetherbackbone and its large conformational freedom, which causes the reduction of overall blood plasma protein adsorption and prevents MNPs agglomeration (**B**). Highly hydrophilic PMSEA [poly(2-(methylsulfinyl)ethyl acrylate] coating turned out to be even more resistant to protein binding as compared to PEG and thereby provides great low-fouling properties (**C**)

One of the most efficient polymers used for MNPs functionalization is polyethylene glycol (PEG) [72], an US Food and Drug Administration-approved macromolecule with different molecular weights. PEG causes the reduction of overall blood plasma protein adsorption and prevents MNPs agglomeration, thus helping MNPs escape from the MPS [73] (Fig. 5B). It has also been shown that PEG coating nanoparticles induces adherence by clustering proteins [74] and consequently makes MNPs unrecognizable to macrophages. PEGylated nanoparticles show a lower cell uptake rate by macrophages [75, 76]. It should be noted that the larger molecular weight or density of PEG, the longer half-life of the nanostructures can be obtained [77]. In addition, PEG has flexible chains that can adopt different conformations. The high intensity and rate of changing these conformations translate into lower probability of plasma protein binding, and thus increase the circulation time in the blood [78].

**Fig. 6:**
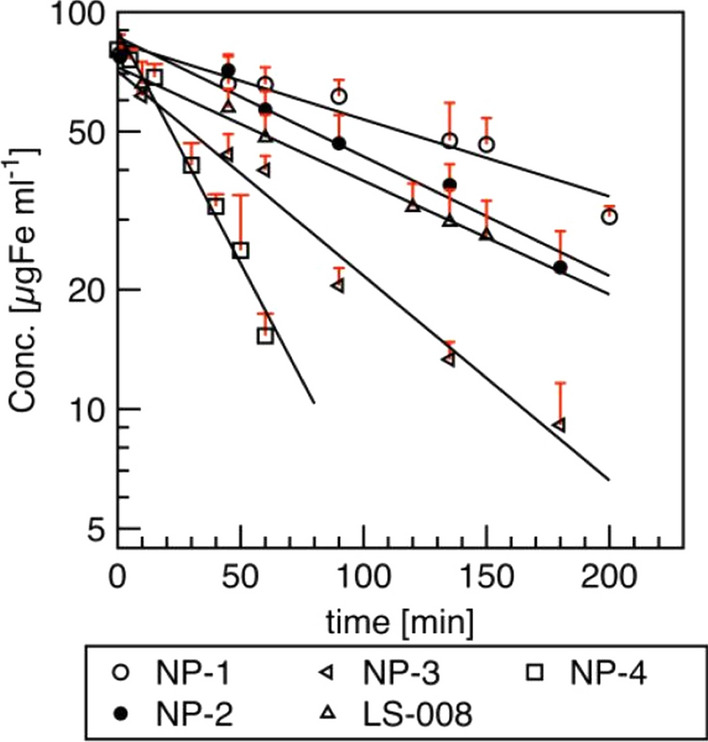
Clearance of MNPs samples from ex vivo Magnetic Particle Spectroscopy measurements; n = 3 per time point. Pharmacokinetic parameters were obtained after fitting data to a first-order elimination model. Republished from Ref. 79 under the terms of the Creative Commons Attribution Licence (CC BY) (http://creativecommons.org/licences/by/4.0/)

There is a large number of reports concerning the effect of PEG coating with specific weight of magnetic nanoparticles on their circulation time and clearance. It was demonstrated that by increasing molecular weight of PEG from a few thousands to a few hundred thousands, the blood circulation time of MNPs was prolonged from 30 min to 24 h by reducing RES uptake. Khandar et al. [79], in turn, tested the pharmacokinetics of magnetic nanoparticles coated with PMAO-PEG-NH_2_ (PMAO – poly(maleic anhydride-alt-1-octadecene)) for various variants of these particles differing in PEG weight and loading percentage. For the same PEG loading density (25%) the half-time decreased with increasing PEG molecular weight (Fig. 6): 5 kDa PEG t_1/2_ = 155 min (sample NP-1), 10 kDa PEG t_1/2_ = 100 min (sample NP-2) and 20 kDa PEG t_1/2_ = 58 min (sample NP-3). *LS-008* MNPs (20 kDa, 18.8% PEG loading density) had the longest half-life among MNPs coated with 20 kDa (t_1/2_ = 105 min), whereas MNPs of sample NP-4 (20 kDa, 12.5% PEG loading) had the shortest half-life t_1/2_ = 28 min. Also Kratz et al. [80] developing MPI tracers obtained magnetic multicore particles (MPCs) modified with PEG of different chain lengths (from 2 to 20 kDa) coupled with amines. The resulting variants: MCP-PEG10K (after one pegylation step) and MCP-PEG10K2 (after a second pegylation step) showed the mean blood-half-lives of 2 and 62 min, respectively. It can therefore be concluded that the increased blood half-life of the second variant was due to the higher PEG density on the particle surface.

Since both the size of MNPs (Sect. 2.2.1.) and the surface PEG modification can prolong the circulation time in the bloodstream, there are competing papers on whether the size or the PEG surface functionalization is a dominant factor influencing the MNPs pharmacokinetics [64, 81, 82]. The effect of the core size and the PEG coating on the blood circulation time was investigated [83]. The authors obtained highly monodisperse MNPs with different core sizes (14 and 22 nm) and coated with phosphorylated mPEG of different molecular weights (2 and 5 kDa). The half-life of 14 nm MNPs@PEG2 was about 15 min, the half-life of 14 nm MNPs@PEG5 was extended to 24 min, whereas the half-life of 22 nm MNPs@PEG5 amounted to 27 min. The results indicated that PEG coating layer rather than the core size influences blood circulation time and tissue clearance.

##### Polyethylene glycol (PEG) conjugates with other molecules

For the nanoparticles coating, PEG conjugates with other molecules are also used. For example, polyethylene glycol (PEG)-conjugated and starch-coated MNPs (PEG–starch–MNPs) for enhanced photothermal cancer therapy (PTT) were developed. The authors found that plasma half-life of PEG–starch–MNPs was 2.7 h, whereas, to compare, t_1/2_ of starch–MNPs was 5.8 min [84].

In the recent report [85], folic acid (FA) conjugated polyethylene glycol (PEG)/polyethyleneimine (PEI)-MNPs nanoparticles loaded with modified paclitaxel (SPTX) (SPTX@FA@PEG/PEI-MNPs) as a drug carrier with beneficial pharmacokinetics was proposed. Paclitaxel (PTX) has interesting anticancer activity, but it is insoluble in water. Therefore magnetic nanoparticles are used as an ideal drug delivery system. Pharmacokinetic results demonstrated that SPTX@FA@PEG/PEI-MNPs exhibited long blood circulation time in rats in vivo (t_1/2_=3.41 h).

MNPs coated with the fourth generation (G4) of polyamidoamine (PAMAM) were synthesized [86]. Surface amino groups of dendrimer molecules were conjugated with mPEG (IONPs-G_4_@PEG) (Mw = 4 kDa) [87]. The studies using the mouse model have shown that the blood iron levels persisted and increased slightly up to 4 h, while after 8 h this amount decreased, which might have been caused by the accumulation of G4@IONPs inside the tissues. At that time, also the amount of iron decreased in the tissues so a hypothesis was put forward that G4@ IONPs was taken up by other tissues such as lymph nodes or the spleen. After 12 h, the amount of iron increased again and then, at the end of 24 h, the value reached approximately half of the initial amount.

Moreover, PEG and Cys-coated ultra small MNPs for angiography and tumor MR imaging applications were developed [88]. The magnetic core MNPs for both Fe_3_O_4_-PEG-Cys and Fe_3_O_4_-mPEG displayed a mean diameter of 3.2 nm and 3.1 nm, respectively. The protein resistance studies have shown that at the same Fe concentration, the Fe_3_O_4_-mPEG nanoparticles absorbed 2–3 times more protein than the Fe_3_O_4_-PEG-Cys MNPs.

Multimodal doxorubicin loaded magnetic nanoparticles for VEGF (vascular endothelial growth factor) targeted theranostics of breast cancer were developed [89]. Magnetic nanoparticles coated with albumin and PEG were combined with monoclonal antibody anti-VEGF and doxorubicin (Dox). The authors tried to optimize two parameters: firstly the size of BSA-coated magnetic nanoparticles used which were less than 50 nm. Secondly, all types of magnetic nanoparticles were coated with PEG in order to provide efficient Dox loading and prevent interaction with plasma proteins. Analyzing the blood circulation process it was observed that magnetic nanoparticles elimination was characterized by two phases: the first component corresponded to fast elimination of magnetic nanoparticles in the first hour and the second component corresponded to the pool of longer circulating magnetic nanoparticles. The blood half-life time of the obtained nanostructures was significantly higher than value of clinically approved dextran coated iron oxide nanoparticles (AMI125) [90]. Thus, the authors developed MNPs with circulation time long enough to be delivered to the tumor and to provide effective binding to tumor cells.

Despite the popularity and the undeniably positive effect of PEG-functionalization nanoparticles on their pharmacokinetics, PEG-coated MNPs also have their disadvantages. Those are especially evident when there is a need for multiple administration of nanoparticles, e.g. when monitoring tumor growth. Some types of PEGylated MNPs had a very high elimination rate by the MPS system after the second injection [91]. This phenomenon is called accelerated blood clearance and the mechanism is suggested to involve the production of anti-PEG IgM antibodies by the spleen after the first administration of the nanoparticles [92].

##### Other synthetic polymers

PLGA poly (D,L-lactide-co-glycolic) acid—based superparamagnetic nanocarriers of DTX (docetaxel) for specific delivery of the drug to breast cancer cells were developed [93]. PLGA is an FDA (Food and Drug Administration) approved biodegradable polymer suitable for clinical applications. However, the research conducted by Qia et al. [94] was particularly noteworthy. Namely, the authors developed iron oxide nanoparticles coated with highly hydrophilic sulfoxide-containing polymer—poly[2-(methylsulfinyl)ethyl acrylate] (PMSEA). It was found that the PMSEA coated MNPs had a more hydrophilic surface than their PEGylated analogues and demonstrated significantly reduced macrophage cellular uptake and much less opsonisation by human plasma proteins (Fig. 5C). In vivo study of biodistribution and pharmacokinetics showed much longer blood circulation (≈ 2.5 times longer with respect to elimination half-life *t*_1/2_) and approx. two times reduced accumulation (in the organs such as the liver and the spleen) for MNPs coated by PMSEA in comparison to those coated with PEG. Thus, PMSEA coated nanoparticles can be a great alternative to PEG-ylated MNPs.

##### Polysaccharides and derivatives

Other frequently used polymers for MNPs coating are chitosan and dextran and their derivatives [95, 96]. Both compounds reduce uptake by RES and increase the circulation half-time.

An interesting form of nanoparticles as potential MRI agents has been proposed: MNPs clustered into raspberry shapes within a polymeric envelope [97]. The self-assembling polymer used was a chitosan amphiphile: N-palmitoyl-N-monomethyl-N-N-dimethyl-N-N-N-trimethyl-6-O-glycolchitosan (GCPQ). It was demonstrated that a positively charged raspberry MNP, comprising 5 nm MNPs clustered into a larger raspberry shape, might be used as a superior MRI negative contrast agent. Clustering, as compared to the synthesis of larger MNPs, means that these 4–5 nm core size MNPs were more amenable to extraction *via* the urine [98]. The pharmacokinetics studies results have shown that the raspberry MNPs had a blood half-life (t_1*/*2_) of 28.3 min, but, what was important, no adverse effects were observed in any of the animals in the experiment, even when the administered dose was several fold-higher than the one needed for MRI.

An important factor that may influence the circulation time of MNPs is the binding strength of coating molecules. If molecules are bound on the surface of nanoparticles by weak, non-covalent bonds, some of them may become detached from the surface of MNPs when injected into the bloodstream [99]. Then a certain fraction of these small, unbound molecules are removed from the body through the urinary system, while the remaining MNPs aggregate and are transported to the liver [100, 101]. The way to overcome this problem is cross-linking of the coating molecules. After this modification, a hydrogel layer is formed on the surface of the nanoparticles, protecting them against opsonization [102]. An example of such a substance is dextran, which binds on the surface of nanoparticles only due to relatively weak hydrogen interactions between the hydroxyl groups of dextran residues and surface oxide hydroxide groups. However, when the dextran molecules become cross-linked with e.g. epichlorohydrin as an alkylating agent, the blood half-life of such nanostructures increases up to 12 h in the mouse model [67, 102, 103].

##### Monomers and small molecules

In many studies inorganic citrate groups have been employed acting as stabilizers for MNPs. It was found that citrate molecules prevented the formation of large aggregates that would be an easy “trophy” for MPS [104] and the citrate stabilization is provided by electrostatic repulsions. The small molecule targeting groups such as citrate groups are not only easy to prepare with their simple conjugation chemistry, but also provide multiple functional groups [105]. In the case of large surfactant molecules and long polymer chains some binding affinity may be lost through steric hindrances, while this is avoided when it comes to small molecules.

Magnetic nanoparticles can be silanized as well, for instance with (3-aminopropyl) triethoxysilane (APTES) to render particles with amine groups, which may then establish various kinds of chemical bonds, thus enabling the immobilization of organic compounds and biomolecules [106]. APTES also provides biocompatibility and stability to the nanoparticles [107]. Furthermore, the larger the aminosilane thickness on the surface of the magnetic core, the longer the circulation of the probes in vivo reported [108]. The authors claimed that a thick layer of aminosilane protected against serum protein adsorption finally leading to the large half-life of the nanostructures: almost 6 h after the injection of nanoparticles 190 nm-wide.

It is very important to mention that circulation time is generally decreased when additional biomolecules such as drugs or cancer targeting agents are attached to the nanoparticles surface [67]. It is caused by the increase in the hydrodynamic diameter of the nanocarrier following the molecules loading. Therefore, attention should always be paid to the optimal amount of the drug to be loaded in order to obtain the desired therapeutic effect.

It should also be stressed that the hydrophobicity of nanoparticles is an important factor in their interaction with plasma proteins and thereby for in vivo circulation time [109]. MNPs with a more hydrophilic surface can significantly minimize the protein absorption and therefore reduce the uptake by the MPS [110]. For example MNPs coated with zwitterionic molecules contain equal numbers of negative and positive groups and therefore have a large number of water molecules surrounded via hydrogen bonding [111]. Therefore zwitterions can form a dense water shell around the MNPs preventing protein adsorption [112].

The characteristics of the commonly used MNPs coating molecules is shown in Table 2.

**Table 2:** The commonly used materials for MNPs functionalization

Organic compounds		
Coating type		Advantages/applications
Monolayers and small molecules	Citrates	Stabilizers MNPs; the free carboxylic groups render a sufficient negative charge on the surface of particles making them hydrophilic
	Folic acid	Effective tumor targeting agent
	Phosphates	Surfactant and stabilizer for nanoparticle dispersion; affords efficient binding ligands on the surface of MNPs
	Amines and aminosilanes	Stabilizing agents in the fabrication of various functionalized MNPs; provide -NH_2_ groups for subsequent functionalization and attaching drugs; used for drug delivery applications
	Thiols	Very high binding affinity towards MNPs; used to functionalize MNPs for targeted drug delivery
Macromolecules	Synthetic polymers	
	Polyethylene glycol (PEG)	Enhances the hydrophilicity and water solubility; provides stability and prevents MNPs agglomeration; PEGylated nanoparticles show a lower cell uptake rate by macrophages, which increases the circulation time in blood; provides -OH groups; used as T_1_ MRI contrast agents and drug delivery systems
	Poli vinyl alcohol (PVA)	Prevents agglomeration, giving rise to monodispersibility
	Alginate	Improves the stability and biocompatibility
	Natural polymers	
	Dextran	Enables optimum polar interactions with iron oxide surfaces, improves the blood circulation time, stability and biocompatibility
	Chitosan	Enhances the biocompatibility and stability; provides functional groups: -NH_2_ and -OH for subsequent functionalization and attaching drugs; hydrophilic; good for drug delivery applications; widely used as non-viral gene delivery system
	Starch	Improves the biocompatibility; good for MRI and drug target delivery
Inorganic compounds		
Silica		Enhances the biocompatibility and stability of the nanoparticles; the mesoporous silica coating is biocompatible and offers high controlled porosity; good for drug delivery applications; useful in the fabrication of multifunctional MNPs
Metals		The most popular approach in this category is the conjugation of Fe_3_O_4_ with gold because of its biocompatibility and multifunctionality; the final applications are numerous: medical imaging (MRI, CT, PA), radiosensitiation, radiofrequency ablation, biosensing, cell sorting
Metal oxides		Metal oxide (ZnO, TiO_2_) functionalization has photocatalytic applications

#### Surface charge

**Fig. 7:**
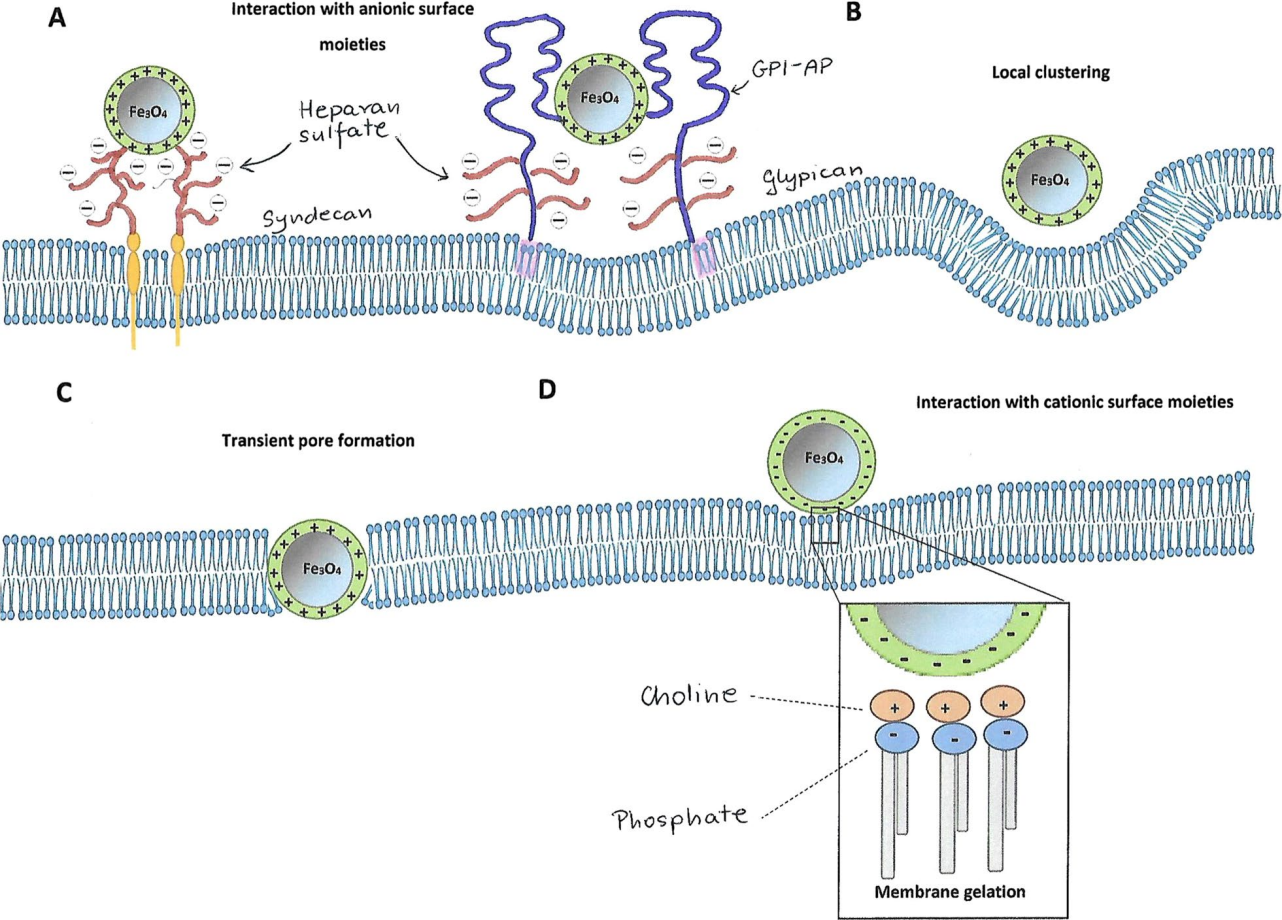
Interactions between positively (**A–C**) and negatively (**D**) charged magnetic nanoparticles (MNPs) and the plasma membrane. Electrostatic interactions with cationic MNPs and anionic syndecans and glypicans containing heparan sulfate (**A**). Nonspecific cationic MNPs interactions with anionic phospholipids (**B**). Transient pore formation by small cationic MNPs (≤ 20 nm) due to the strong attraction to the inner membrane layer in phosphatidylserine-rich regions (**C**). Local membrane gelation induced by anionic MNPs in phosphatidylcholine-rich membrane microdomains (**D**)

The surface charge of nanoparticles has a great influence on the interaction with the cell membrane due to the many charged membrane components such as phospholipids, glycolipids and proteins. Cationic nanoparticles in particular are strongly attracted by negatively charged phospholipid residues and a few proteins [113]. For example, syndecans are single transmembrane domain proteins which carry three to five heparan sulfate and chondroitin sulfate chains. In other words they are proteoglycans, allowing interaction with a large variety of ligands and contributing to cationic MNPs endocytosis [114, 115] (Fig. 7A). The second major group of the proteoglycans are glypicans – peripheral proteins that can also mediate positively charged MNPs endocytosis through lipid raft-dependent mechanisms [114–117] (Fig. 7A). In addition to the above listed mechanisms, there also occurs the nonspecific cationic MNPs binding through electrostatic interactions with phospholipids promoting their local clustering [117] (Fig. 7B). In the case of smaller cationic nanoparticles (< 20 nm), the electrostatic interactions can lead to the formation of transient pores enabling MNPs translocation through the membrane [114] (Fig. 7C). This phenomenon is influenced by the strong interaction of small cationic nanoparticles with the inner side of the membrane, which is richer in negatively charged lipids [118, 119].

In contradistinction to cationic MNPs, anionic nanoparticles are only internalized by the endocytic mechanism. Due to the repulsive interactions with the membrane components, they show much lower affinity for it and thereby have much lower internalization rates [120]. The detailed mechanism of the anionic MNPs endocytosis is not well understood yet, however there are reports suggesting that their uptake takes place by promoting local changes in plasma membrane. Wang et al. [121] explained that the interaction takes place due to the presence of phosphatidylcholine in the membrane, which contains dipoles of phosphate and choline (P^−^–N^+^). Anionic nanoparticles can interact with the N^+^ terminus and cause a slight hollow in the membrane, which has been observed to transduce into local membrane gelation (Fig. 7D). This, in turn, may initiate the endocytosis process.

Anionic nanoparticles, just as cationic ones, may also interact with membrane components in an non-specific way, i.a. through transmembrane proteins known as SR-As (class A scavenger receptors). These proteins possess cysteine-rich side chains able to recognize anionic particles, including those coated with dimercaptosuccinic acid (DMSA) [122] and carboxydextran [123].

Despite the fact that negatively charged nanoparticles are recognized by cells, including phagocytic ones, to a much lesser extent than positively charged nanoparticles, neutral nanoparticles interact with the membrane even less than negative particles [124]. For example, the half-life of the neutral Ferumoxtran-10 (nanoparticles coated with dextran and of 35 nm in hydrodynamic diameter) was much longer (24–36 h) than that of anionic Ferumoxytol (the same coating time and similar d_H_, 10–14 h) [125, 126]. The neutral MNPs can also be cleared through the urinary tract [127].

A high negative value (for example – 35 mV) of the MNPs surface minimizes the tendency to agglomeration of nanoparticles and thereby the tendency to absorb plasma proteins providing prolonged circulation time to an extreme degree [128].

Since the charge of the nanoparticles’ surface has an impact on the degree of the proteins’ absorption and circulation time in the bloodstream, the types of functional groups displayed on the MNPs surface play an important role in determining the half-life of the nanostructures [129]. For example, MNPs with a large number of amino groups are expected to have a positive charge, whereas hydroxyl, sulphate and carboxyl groups usually contribute to a negative charge. The studies regarding the direct role of functional groups on the MNPs pharmacokinetics are still in progress, however.

There are many reports on the influence of the nanoparticles charge on their circulation time. Some of them appear to be inconsistent, but probably the reason of this variance is that the pharmacokinetics of nanoparticles is a combination of many factors, such as the size, shape, type and density of the covering materials, and many others. Ergo et al. [130] studied positively and negatively charged dextran-coated nanoparticles with a diameter of 26 nm using the rat model. The study results indicated that the positively charged MNPs were mainly retained in the liver and had a rapid clearance time (2 min). The negatively charged nanoparticles were accumulated in lymph nodes and showed longer clearance (50 min). Conversely, uncharged (neutral) nanoparticles showed the longest circulation time, and their uptake in the liver and the spleen was significantly lower than that of charged particles [131]. In another study, the influence of PEG-oligocholic acid based micellar nanoparticles surface charge on their biodistribution was explored [132]. The results showed a high liver uptake for high positively or negatively charged MNPs, while slightly negative particles had a very low liver uptake. Zelepukin et al. [51] compared the circulation of 100-nm uncoated anionic (UC/A) and cationic (UC/C) nanoparticles, and 1-µm COOH- and NH_2_-coated polystyrene beads. In both cases, an increase of the negative charge of the particles prolonged their circulation. On the other hand, it was reported that MNPs of different sizes coated with PVP and with zeta potentials ranging from + 12 to + 14 mV were accumulated in the liver to a similar degree as Feridex (d_H_=58 nm and – 25 mV) [133].

#### Technical aspects

Not only do the characteristics of nanoparticles affect the time of their circulation, but also some technical factors may contribute to the pharmacokinetics of MNPs. The rates of the particles’ uptake may vary among the different strains of mice due to the differences in the genetically predetermined immune profiles [51]. Diverse pathologies and diseases may also influence the immune system and, indirectly, the behaviour of nanoparticles. It has been reported that macrophages in BALB/c mice eliminate 50-nm particles 2.3 times faster than in C57Bl/6 mice [51]. It has been also shown that tumor growth in vivo affects the pharmacokinetics of the nanoparticles administered. The authors found that in the case of 50-mm^3^ tumors, nanoparticles exhibited 20–30% decrease in half-life time, while with 250-mm^3^ tumors − 5.2-fold and 2.5-fold reduction for B16-F1 and EMT6/P tumors, respectively. Therefore, the pharmacokinetics of nanoparticles acting as drug carriers may vary significantly with cancer progression. Moreover, the authors suggested that the particle circulation can be influenced by many other diseases, mainly those associated with the functioning of the immune system [51].

There are also reports indicating that the circulation time of the nanostructures depends on the dose administered. For example: male rats were intravenously injected with the following formulations individually: MNP-alginate at a dose of 6.12 mg Fe/kg (SPIO-low dose) and MNP-alginate at a dose of 12.23 mg Fe/kg (MNP-high dose) [134]. The results showed that SPIO-alginate was eliminated at a high rate from the serum (half-life of 0.27 h) at a dose of 6.12 mg Fe/kg and accumulated mainly in the liver and the spleen after injection, whereas the t_1/2_ of MNP-alginate at a high dose was 0.59 h. Prospero et al. [135] investigated the circulation time of citrate coated magnetic nanoparticles in rats depending on their mode of administration. Six animals received three injections of 300 µl of nanoparticles at 35 min intervals, whereas the other six animals received only one dose of 900 µl of the same MNPs. The results showed that t_1/2_ significantly increased after each subsequent dose. The following were found: t_1/2_ of 11.5 min, 20.3 and 24.7 min for the first, second and third injection, respectively, in the first group. The t_1/2_ obtained for the single administration in the second group was 46.7 min, which was statistically different from each administration in the first group. The effect of the administered MNPs dose (100 nm glucuronic acid coated MNPs) has been also studied [51]. When the administered doses were less than 1 mg (50 µg/g tissue), the half-life time was almost constant at the level of 1–1.6 min. Upon the further increase of the administered amount, the particle circulation was prolonged up to 45 ± 14 min for the 10-mg dose. This effect may have been caused by the overloading of the entire mononuclear phagocyte system, which were not able to eliminate such large doses of the particles. On the other hand, the adjustment in MNPs pharmacokinetics during multiple nanoparticle administrations was tested. For comparison purposes, a single dose was administered in the second series. Multiple subsequent administrations of the particles resulted in the prolonged circulation time, starting from the second injection, and each new dose intensified the effect. However, after one-day break between the injections, no significant changes in the circulation time of the particles were observed in comparison to single MNPs injection.

## Pharmacokinetics and clearance of MNPs in other administration methods

Inhalations of nanoparticles are usually administered for imaging and treatment of lung diseases [136]. In this way the nanoagents reach the lung alveoli [137], where macrophages phagocytize MNPs. The intranasal way of administration is also suggested as a way to deliver nanoagents to the brain [138], however the results of the studies in this field are still controversial and the mechanisms of the crossing the blood-brain-barrier (BBB) through this method are still investigated [139, 140]. In the case of brain tumors, such as glioblastomas, treated with magnetic hyperthermia therapy, direct intratumoural injection is the primary method of MNPs delivering [141]. Intravenous administration is then avoided due to blood-brain tumor barrier (BBTB), which is admittedly more permeable than the healthy BBB, but is still very selective and impenetrable to many chemotherapeutic agents.

Nanoparticle characteristics such as size, charge or coating molecules play an important role in their pharmacokinetics in the lungs. As is turned out, almost 90% of the naked MNPs dosage (with the size of 20–30 nm in diameter) were still present in the lung even two weeks after the administration [142]. On the other hand, negatively charged cross-linked MNPs with hydrodynamic diameter of 36 nm were cleared from the lungs 3 h after administration [143]. Other studies have shown that after 28 days of silica coated MNPs (d_H_=50 nm) inhalation, a significant amount of them was accumulated in the liver, kidneys and testes, whereas the percentage of the nanoparticles remaining in the lungs was similar to other tissues (for example the heart or the brain) [140, 144]. Unquestionably, further research is needed to elucidate the mechanisms of pharmacokinetics, biodistribution and any contradictions regarding nanoparticles administered intrapulmonary.

When it comes to the oral administration of the MNPs, there are several biological barriers that must be overcome in order to successfully deliver therapeutic agents immobilized on the nanoparticles. For example, digestive acids and enzymes can easily destroy some nanoagents. Suitable coatings molecules (such as casein protein or silica) with pH_a_ values lower than 3–5, however, are able to protect them against degradation [145]. On the other hand, it has been experimentally confirmed that the model acidic drugs immobilized on MNPs exhibited significantly higher solubility at pH corresponding to the environment prevailing in the initial sections of the digestive system in comparison to the unbound drugs [146]. This ensures a much greater effectiveness of the immobilized drugs at a given dose.

The second difficulty in oral administration is the need to pass the transport barrier of the intestinal epithelium, which can be reached by conjugation MNPs with the agents being permeation enhancers. The example are peptides that specifically bind to FcRn receptors in intestine epithelial layer [147]. Next, MNPs can cross the liver sinusoids and then gain entry to the main blood circulation system. Thus, the liver is the major clearance organ in the oral administration of nanoparticles, unless special MNPs surface modifications make them resistant to these macrophages. The magnetic nanoparticles remaining in the digestive tract are extracted through the feces [148].

In conclusion, after the oral administration, nanostructures should be absorbed through the epithelium and then enter the blood circulation. As particle size decreases, the contact with epithelial surfaces increases resulting in a higher uptake of MNPs [149]. Thus, many physical, chemical, and biological properties such as surface modifications, particle size, and intestinal contents affect the bioavailability and absorption of the swallowed nanomaterial [150].

Other administration methods, such as intraperitoneal [151, 152] or intra-muscular and subcutaneous [153, 154], have also been proposed as an alternative for the above described routes. However, careful research is still needed to determine the long-term distribution and clearance of the MNPs injected by these methods.

## The methods of determining the pharmacokinetics and biodistribution of MNPs

The half-life of nanostructures is a factor of major significance for in vivo experimentation and clinical applications [155, 156]. The ability of in vivo monitoring of MNPs distribution and clearance is the goal of the researchers and clinicians [157]. The evaluation of the MNPs half-life is usually based on multiple measurements of their concentrations using techniques such as Electron Spin Resonance (ESR) [158, 159]. ESR is commonly used to characterize the physical properties of various nanomaterials,^,^ including functionalized magnetic nanoparticles. The technique can be applied to observe the differences resulting from the interaction between the material surface and environment. These methods, however, only provide results at specific time points, while developing the techniques that would enable real-time in *vivo* detection remains a challenge [160].

The methods used to detect MNPs concentration in blood and organs can be generally divided into imaging, spectroscopy and magnetometry techniques. Imaging methods for determining the biodistribution of nanoparticles include, among others, Transmission Electron Microscopy (TEM) which is characterized by a high magnification and resolution. This technique enables the detection of nanoparticles distributed in intracellular and extracellular thin fragments of tissues [161]. Moreover, TEM is related to elemental analysis [162], allowing to differentiate nanoparticles located in different structures, such as ferritin or lysosomes of the liver and macrophages of the spleen. Thereby, TEM provides information on biodistribution and MNPs degradation ways in the organism [25]. The disadvantage of TEM is the need for costly preparation procedures. Furthermore, the technique provides information only from specific, very limited areas of tissue.

Histology, which involves detection using an electron microscope, provides information on the distribution of magnetic nanoparticles in larger areas of isolated tissues. Customarily, selected tissue fragments are stained with Prussian Blue dye. The method is more economically advantageous than the TEM technique, but it also has its limitations. Firstly, histology may not detect MNPs prior to degradation [163] and secondly, it does not distinguish between endogenous iron and that which has been injected.

In addition to the ex vivo imaging techniques depicted above, the Magnetic Resonance Imaging (MRI) method can be applied in order to image MNPs distribution in vivo. Depending on the magnetic properties of the studied particles, it considers both relaxation pathways: the first structures are those that reduce the longitudinal (T_1_) relaxation time and cause positive contrast enhancement (Gd^3+^ complexes), and the other group includes particles called negative contrast agents which are based on magnetic iron oxide nanoparticles resulting in a darker condition in the T_2_-weighted image [164, 165]. Still, it has been reported that Fe_3_O_4_ nanoparticles with a size smaller than 5 nm have decreased magnetic moment which causes strong T_2_ suppression effect [166]. Consequently, ultrasmall MNPs can be used for T_1_-weighted imaging [167]. Also, when a pulse sequence with an ultrashot time echo (UTE) is used, the T_2_ effect is overcame and MNPs can be exploited as T_1_ agents [168]. Generally, MRI is an noninvasive and repeatable method for determining the blood circulation time of contrast agents, especially in small animals (mice, rats).

Magnetic Particle Imaging (MPI) is an in vivo imaging method which makes use of the nonlinear magnetic response of magnetic iron oxide nanoparticles [169, 170]. MPI exploits the combination of an alternating excitation magnetic field and a static magnetic field gradient for real-time. The technique provides prominent contrast and signal-to-noise ratio because the signal is deprived of background. The selected region can be rapidly and continuously detected for real-time imaging of MNPs distribution. It can be postulated that MPI combines the safety of Magnetic Resonance Imaging [171] and the sensitivity of Positron-Emission Tomography (PET) [172]. However, the MPI method is still under extensive investigation. An important issue is to develop appropriate MPI tracers. Eberbeck et al. [173] studied the effect of size distribution on MPI performance and demonstrated that only 30% of the T_2_-MRI contrast agent Resovist® contributed to the MPI signal, whereas the remaining 70% was practically unresponsive to MPI. Khandhar et al. [79] studied the pharmacokinetics and biodistribution of PMAO-PEG-coated MNPs using MPI for various variants of these particles differing in PEG weight and loading percentage. The results showed that the MPI tracer called *LS-008*, coated with 20 kDa mPEG-NH_2_ at 18.8% of loading capacity, was the most stable and had a blood half-life of 105 ± 10 min in mice. In vivo MPI imaging of mice revealed the intravascular signal persisting for up to 3.5 h after the injection. These results suggested that *LS-008* is an all-purpose tracer for blood MPI, with potential applications in cardio- and cerebrovascular imaging. Moreover, Keseleman at al [174] investigated biodistribution and clearance using *LS-008* and Ferucarbotan - multi-core carboxydextran-coated magnetite nanoparticles, as MPI tracers. In animals injected with Ferucarbotran, most of the tracer cleared into the liver immediately following the injection, which would make it a great tracer for imaging the liver. On the other hand, the *LS-800* particles remained in the blood for several hours after the injection and then cleared into the spleen. This is especially useful for MPI in applications such as angiography [175], cancer imaging [176] or therapeutic applications [177] where long circulation time is desirable.

Magnetic Particle Quantification (MPQ) technology was developed by Zelepukin et al. [178] for extensive study of magnetic particles blood circulation. The low invasiveness and high resolution of this technique allowed the authors to study the influence of various factors on the MNPs kinetics in the blood. They reported that the circulation time of nanoparticles was influenced not only by their size and surface chemistry, but also by the method of administration and the animal model. In this method, nanoparticles are excited by magnetic field waves at two frequencies f_L_ and f_H_ with two amplitude H_L_ and H_H_, respectively. The response is measured at combinatorial frequencies fi = n×f_H_ ± m ×f_L_, where m and n are integers. The values of m and n may vary for the signal-to-noice ratio (SNR) to be the most optimal. The earlier studies showed that the MPQ technique allowed to measure very low amounts of the nanoparticles without destroying the sample [179]. Moreover, the low amplitude and frequencies used in MPQ protected the MNPs from heating and agglomeration which normally occurred when interacting with a magnetic field. Detectors correlated with the MPQ technique are successfully used in cytological and bioanalytical research [180].

The AC Susceptibility (ACS) technique consists in measuring the magnetic moment of a sample which is exposed to an oscillating external magnetic field [181]. In a typical measurement setup the static field is provided by the permanent magnet, and the lock-in amplifier drives a modulation (or primary) coil to generate an AC magnetic field and produce a time-dependent magnetic moment in the sample. The susceptometer features two pick-up coils symmetrically positioned with respect to the primary coil. One coil contains the sample, whereas the other one is wound in the opposite direction and serves as the reference coil. After a magnetically susceptible material is exposed to an external magnetic field H, the resulting magnetic field will be B = µ_0_ (H + M) 0, where µ_0_ is the magnetic permeability in a vacuum, B is the magnetic induction or B-field, H is the externally applied magnetic field strength, and M is the magnetization field from the magnetic material. The magnetization field arises from the magnetically susceptible material, where M = Hχ_v_ and χ_v_ is the volume magnetic susceptibility, which is > 0 for paramagnetic materials. While the M-field is generated only inside of the magnetic material, it creates an additional external B-field that contributes to the magnetic field detected by a sensor. Only the signal induced by the sample’s magnetization is measured and all other background contributions are subtracted.

The ACS technique can be used to distinguish heterogeneous nanoparticles in the imaged tissue fragment and to image MNPs tracers inside tumors [182]. Moreover, in the case of AC Susceptibility Imaging (ASI), the maximum contrast for specific types of nanoparticles can be selected by choosing specific frequencies. Additionally, it was demonstrated that ACS provided information on the MNP coating and agglomeration process which could not be investigated with the DLS technique due to the additional presence of non-magnetic polymers in the suspensions [183]. It has been also shown that AC Susceptibility technique can be successfully used for monitoring the degradation of magnetic nanoparticles in biological media [184].

Inductively Coupled Plasma (ICP) techniques coupled with Atomic Emission Spectroscopy (ICP-AES) [185, 186] or Mass Spectrometry (ICP-MS) [187, 188] also find their application in the detection of nanoparticles. These systems use electromagnetic induction to generate argon plasma at the temperature range of 6000-10000 K. As a result, the molecular and ionic bonds are broken in the sample. In the case of ICP-AES, sample atoms are excited by the plasma, and the electromagnetic radiation emitted by atoms is quantified by a spectrometer. In ICP-MS, the plasma causes ionization of the sample, and the mass-to-charge ratio is recorded by a mass spectrometer. It should be noted, however, that the ICP techniques are destructive methods as they are based on determining the amount of elemental iron in digested tissues. Moreover, similarly to Prussian Blue staining, they do not allow for the differentiation of the endogenous and administered iron [189]. On the other hand, there are reports that the methods enable the detection of iron present in tissues at very low concentrations (nanomoles of iron per gram of the tissue) [189].

Electron Paramagnetic Resonance (EPR) is a sensitive technique for studying iron oxide nanoparticles and free radicals [190]. The basis of this method is the interaction between the external magnetic field and magnetic moments of unpaired electrons in a sample. Gobbo et al. [191] successfully conducted biodistribution and pharmacokinetic studies of MNPs with the use of this technique. Ferromagnetic Resonance Spectroscopy (FRS) is also a suitable technique to quantify magnetic nanoparticles in biological samples [192]. Magnetic Susceptibility Measurement (MSM) [25] and the technique with the use of Superconducting Quantum Interference Device (SQUID) [193] are magnetometry techniques exploiting the magnetic properties of iron oxide nanoparticles for detection.

The detection of magnetic nanoparticles Fe_3_O_4_ is also performed by labeling MNPs by radioactive atoms (e.g. ^59^Fe [194], ^111^In [195] or ^51^Cr [196]) or fluorescent molecules called fluorophores [197, 198]. One of the most often used fluorophores is Cy5.5 - a far-red (and near-infrared) emitting dye. For example, Lee et al. [24] reported a fast and economical method for assessing serum half-life, biodistribution and in vivo stability of chitosan-coated iron oxide Fe_3_O_4_ nanoparticles labeled with the NIRF, Cy5.5.

To sum up, there is a broad range of methods for determining the half-life and biodistribution of magnetic iron oxide nanoparticles, but despite this, research on broadening the spectrum of techniques used is still ongoing. The main goal is to ensure that the method used allows for the continuous detection of MNPs in real time, and is concurrently sensitive, non-invasive, unsophisticated and economically beneficial. The summary of advantages and disadvantages of the most important techniques for determining the pharmacokinetics of MNPs can be found in Table 3.

**Table 3:** Basic techniques used for the characterization of magnetic nanoparticles

Technique	Advantages	Drawbacks	Refs.
* Imaging techniques *			
Transmission Electron Microscopy (TEM)	- Detection of nanoparticles distributed in intracellular and extracellular thin fragments of tissues - Differentiation nanoparticles located in different structures - Information on biodistribution and MNPs degradation ways in the organism	- Costly preparation procedures - Information only from specific, very limited areas of tissue	[161, 162]
Magnetic Resonance Imaging (MRI)	- Noninvasive and repeatable method - Visualizing and distinguishing individual soft tissue - Used in examinations of practically the entire body - Possibility of continuous imaging of moving objects in real time	- Necessity of application a very strong magnetic field - Quite expensive technique	[164−168, 171]
Magnetic Particle Imaging (MPI)	- Prominent contrast and signal-to-noise ratio - The selected region can be rapidly and continuously detected for real-time imaging of MNPs distribution	- Necessity to develop and apply the appropriate MPI tracers	[79, 169, 170, 173–177]
*Spectroscopy techniques*			
Electron Spin Resonance (ESR)	- Characterization of physical properties of various nanomaterials - Observation the differences resulting from interaction between the material surface and environment - Differentiation between the endogenous and administered iron	- Results for only specific time points - The necessity to section the tissue samples in to 2 mm^3^ cubes to fit in the thin ESR glass tubes	[158–160]
Inductively Coupled Plasma (ICP) techniques coupled with Atomic Emission Spectroscopy (ICP-AES) or with Mass Spectroscopy (ICP-MS)	- Detection of iron present in tissues at very low concentrations	- Destructive methods - No differentiation between the endogenous and administered iron	[185–189]
Electron Paramagnetic Resonance (EPR)	- Sensitive and nondestructive method which results in a direct measurement of the MNPs not requiring further data analysis - Performed at low magnetic fields and frequencies, offering the advantage that a much larger sample volume can measured at room temperature - EPR can be combined with MRI which benefits among others in cell tracking studies	- Limitations of the method result from the instability of paramagnetic centers in the tested substances and the reduced sensitivity of their detection for samples containing water	[190, 191]
Ferromagnetic Resonance Spectroscopy (FRS)	- Powerful method for the quantitative determination of internal fields in ferro- or ferrimagnetic materials and nanostructures - Shape of the FMR spectrum contains valuable information about the internal fields in the sample	- Structural information cannot be obtained in a straight-forward way from spectra	[192]
Alternating Current (AC) Susceptibility (ACS)	- Non-invasive method - Tissue sample preparation is minimal and no separation or isolation procedures are needed for the simultaneous quantification of several iron-containing species - The large amounts of tissue can be characterized each time so that representative results are easily obtained	- The need to use ex vivo samples - Time, costs and the relatively low availability of these type of instruments	[181–184]
*Magnetometry techniques*			
Magnetic Susceptibility Measurement (MSM)	- A fast and easy method to quantify MNPs in convenient and accurate way in different media - There is no need of any preliminary modification of the samples - MSM values are only influenced by the iron from magnetic particles and not by free iron in solution	- The same magnetic particles for the calibration and experiments must be used, magnetic susceptibility being sensitive to the size of the magnetic core	[25]
Technique with the use of Superconducting Quantum Interference Device (SQUID)	- Very sensitive technique - These instruments are used in MRI and magnetoencephalography (MEG) for recording the very weak fields, which are produced by electrical currents flowing in the brain’s neural networks	- The noise level is determined by environmental sources, except in those experiments where the SQUID and its signal source are enclosed in a superconducting shield	[193]
Magnetic Particle Quantification (MPQ)	- Method offers highly sensitive, room-temperature and rapid quantification of nanoparticle–cell interactions - The low invasiveness and high resolution - Possibility of measuring very low amounts of the nanoparticles without destruction of sample - Llow amplitude and frequencies used in MPQ protect the MNPs from heating and agglomeration	- Necessity to use only MNPs with nonlinear magnetization - MPQ method cannot distinguish the processes of particle dissolution, transformation of iron oxides to biological forms of iron, excretion of particles from the organism, etc.	[178–180]

## Nonstandard methods of extending the circulation time of nanoparticles in the blood

The preceding chapters present the properties and characteristics of magnetic nanoparticles, as well as technical factors affecting the circulation time of MNPs. Still, a number of unusual methods have also been developed to extend the half-life of nanostructures. For example the so called “cell hitchhiking”, especially RBC hitchhiking, has been extensively studied for the last few decades, and has been shown to efficiently prolong circulation time and increase the targeting of drugs and nanoparticles [199, 200]. Antonelli et al. [201] proposed a strategy of MNPs contrast agents loading in erythrocytes, which enabled them to escape the MPS system thereby prolonging circulation time. The authors used commercially available ferrofluid SHU 555 A, that is magnetic iron oxide nanoparticles coated with carboxydextran sized 60 nm in diameter. The encapsulation of MNPs in erythrocytes was possible due to the fact that red blood cells have sufficiently large pores (50–200 nm) in the cell membrane, which in turn leads to the penetration of nanoparticles [202]. The results of in vivo experiments on mice have shown the biological half-life of MNPs within the loaded RBC fraction of the whole blood was of approx. 12 days, whereas an equivalent amount of free SHU 555 A suspension injected in mice entirely vanished within 24 h. In the following years, the authors presented a comparative study on the encapsulation effectiveness of different types of magnetic nanoparticles in erythrocytes, both commercially available (SHU 555 A, AMI 227 and PMP-50) and newly synthesized, dextran or carboxydextran coated and within range of 30 to 60 nm in diameter, in order to improve the fabrication of new carriers that could be useful in MRI biomedical applications [203]. The results showed that the tested nanoparticles can be successfully loaded into erythrocytes. In later studies Antonelli et al. [204] reported precursory results regarding the RBC loading procedure to the ferucarbotran nanoparticles. Moreover, the authors carried out in vitro administration of human ferucarbotran-loaded RBCs to human macrophages in order to study their interaction with macrophage surface. In brief, 3 µmol Fe were administered either as free ferucarbotran or as ferucarbotran-loaded RBCs. The results of the study consisting in T_1_ NMR measurements on blood samples showed that R_1_ relaxation rate of circulating blood of mice treated with ferucarbotran-loaded RBCs was approximately 1.5-fold higher than blood R_1_ of mice treated with free ferucarbotran, whereby the half-life of bulk ferucarbotran nanoparticles was less than 1 h, whereas blood half-life of ferucarbotran loaded RBCs was 48 h.

In recent years cell membrane-based nanosystems have engaged attention due to their superior biocompatibility and functionality [205]. MNPs coated with biomimetic cell membranes such as stem cells and morphotic elements of blood can increase blood circulation time and accumulation at the tumor site [206, 207]. For example, Bu et al. [208] fabricated and investigated a platelet–cancer stem cell (CSC) hybrid membrane-coated iron oxide magnetic nanoparticles. Their studies showed that the membrane-coated MNPs demonstrated good blood biochemistry, circulation time and histology as well as higher tumor accumulation [208].

**Fig. 8:**
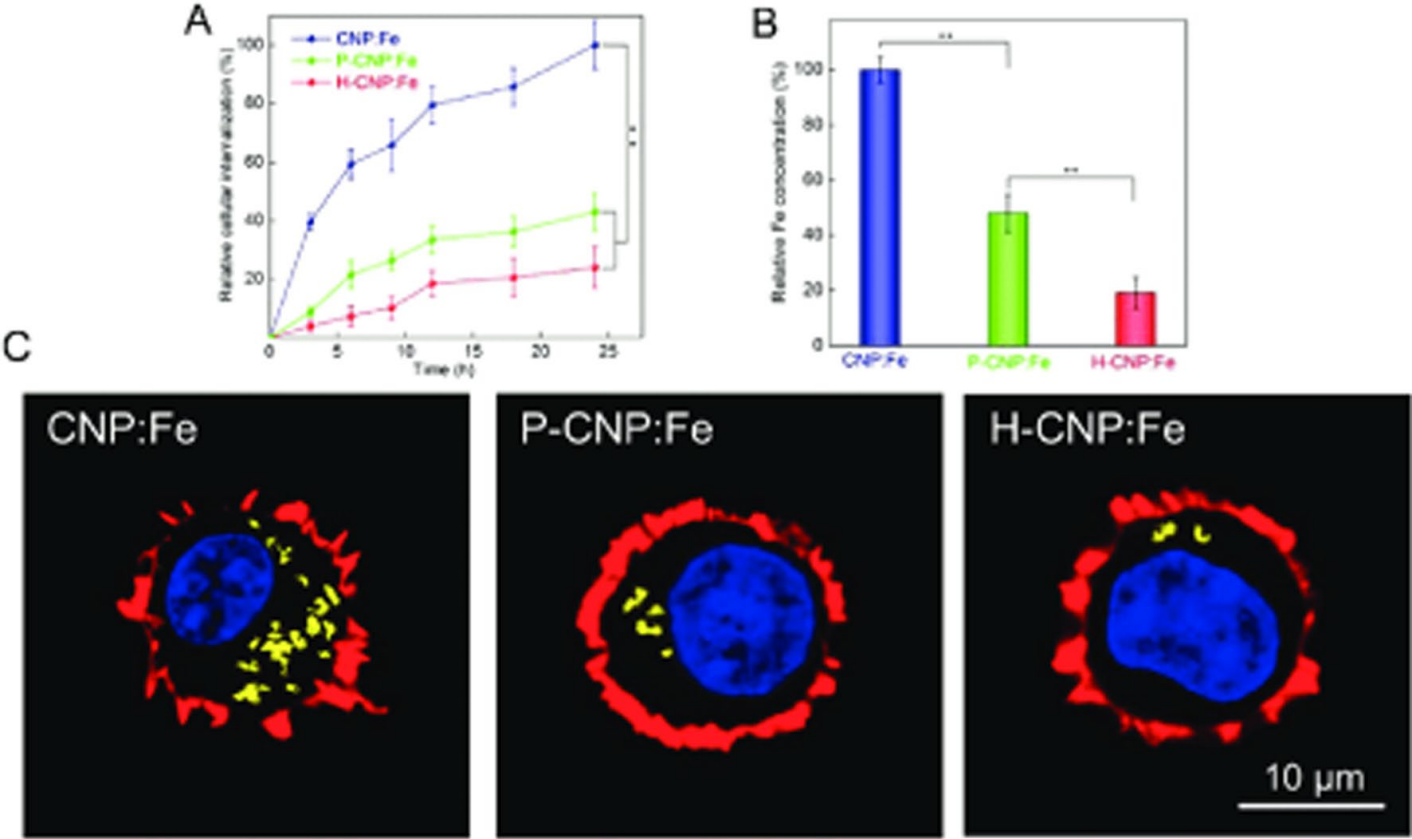
Time-dependent internalization profiles of CNP:Fe, P-CNP:Fe, and H-CNP:Fe internalized by J774A.1 macrophages (**A**). Comparison of intercellular Fe concentration of J774A.1 macrophages after 24 h incubation with different NPs (**B**). The CLSM images of J774A.1 macrophages after incubation for 24 h with different NPs (**C**). Republished from Ref. 209 with permission of Royal Society of Chemistry

Erythrocytes can circulate in the bloodstream for up to 120 days since they have a variety of self-markers on their surface. The new studies indicate that MNPs coating with erythrocyte membrane can significantly extend their blood circulation time. Chemotherapeutic drugs - paclitaxel (PTX) and doxorubicin (DOX ) co-encapsulated into O-carboxymethyl chitosan (CMC) nanoparticles (CNPs) were further camouflaged with erythrocyte membrane by mixing with hemosome (H) (H-CNP:Fe) [209]. The “stealth” performance of the fabricated erythrocyte membrane nanocarriers was compared with the PEGylated ones. The results have shown that the primal CNP:Fe were rapidly internalized by macrophage cells, whereas a decreased cellular uptake rate was observed in the presence of PEG (P-CNP:Fe). As it turns out, after CNPs were masked with erythrocyte membrane, cellular uptake rate become much slower (Fig. 8A). Analogous results were found by the ICP analysis of intracellular Fe content (Fig. 8B). Moreover, the studies using confocal laser scanning microscopy (CLSM) images (Fig. 8C) demonstrated that the slightest fluorescence signal was detected in the case of nanoparticles coated with membrane (H-CNP:Fe). The above results indicate that coating MNPs with erythrocyte membrane can significantly extend their blood circulation time, even more effectively than the traditional PEGylation method.

It was also shown that myeloid-derived suppressor cell (MDSC) membrane-coated MNPs demonstrated their excellent performance in immune escape and MRI imaging [210]. Moreover, in terms of tumor targeting, they exhibited higher efficiency even when compared to erythrocyte membrane coated MNPs. MNPs coated with different types of cracked cancer cell membranes (CCCM) were also developed [211]. It was proven that such nanosystems exhibited self-recognition and highly tumor-selective targeting to the homologous cancer cells in vivo even while competing with another heterologous tumor.

Another approach aimed at extending the circulation time of nanoparticles is blocking the MPS function in vivo by injecting large doses of organic and inorganic materials such as colloidal carbon [212], dextran sulphate [213], methyl palmitate [214], liposomes [214, 215] and fat emulsions [216]. MPS blocking consist in a temporary reduction of macrophage activity due to the uptake of blocking agents mentioned above. As a result, therapeutic or diagnostic particles injected after administration of blocking agents are not so quickly eliminated from the bloodstream by macrophages and thereby circulate longer. It should be noted, however, that the disadvantage of this approach is the need to typically use very high doses of blocking substances [217]. On the other hand Nikitin et al. [218] presented a different approach for efficient MPS blockade that involves a significantly lower dose of a foreign substance: 1.25 mg kg^− 1^ (2–3 orders of amount less compared with previous methods) of allogeneic anti-erythrocyte antibodies which increased the circulation half-life of a range of nanostructures by up to 32-fold. The antibodies intensified the clearance by MPS of its own intact blood cells, particularly erythrocytes. Hence, the authors called this approach MPS-cytoblockade or MPS-erythroblockade. The researchers used a mouse model and a mouse monoclonal anti-mouse-RBC antibody—IgG2a 34-3 C. After the antibody injection, the model tracer: 100 nm fluidMAG-ARA particles (magnetic cores coated with polysaccharide matrix with terminal glucuronic acid groups) were administered. It was found that at 12 h after antibody injection, the 9.8-fold increase of MNPs circulation half-life was achieved. Thereafter, the half-life values have returned fully to the values measured in the absence of the blocking agent, which indicated that MPS-cytoblockade had no long-term effects on the MPS functionality.

Extensive research on the influence of various factors on the circulation time of nanoparticles in the blood under the blocking conditions of the MPS system was carried out [219]. The authors used silica nanoparticles (SiO_2_ particles) with diameters of 100, 250, 500 nm and 1 μm as blocking agents. Firstly, the mice were injected with a large dose of blocker particles (5 mg). Then, following the complete removal of the blocker nanoparticles from the bloodstream, the mice were injected with a small dose (200 µg) of tracer nanoparticles. The administration of 5 mg of the blocker per mouse caused prolongation of the circulation times of the 100-nm tracer MNPs by 3.2 times due to a reduced MPS activity. Next it was examined how the size of the tracer MNPs affects their half-life in the blood under the MPS blockade conditions. It was reported that the MPS blockade induced by 5 mg of 500-nm SiO_2_ particles prolonged half-life of 50, 100, 200-nm tracer particles by 3.9, 3.2 and 4.6 times, respectively. Afterwards, the authors investigated the dependence of the blockade efficiency on the blocker agent size. The tracer nanoparticles half-life increased by 1.5, 2.4, 2.8, and 2.5 times for the 100-, 250-, 500- and 1000-nm blocker, respectively. Thus, it was concluded that the 500-nm blocker nano-agents caused the greatest MPS inhibition. When they blocked macrophages using the 500-nm particles with negative (-67 mV) and positive (+ 27 mV) zeta potentials, they did not observe a significant difference in pharmacokinetics of the tracer particles, whereby the blood circulation half-life increased 2.4-fold with the positively charged blockers and 2.8-fold in the case of negatively charged ones. The researchers also examined the effect of the mice strain on the efficiency of MPS blockade induced by the uptake of nano-agents The circulation times of the tracer agents under the MPS blockade with 500-nm SiO_2_ particles were increased by 3.4, 4.2 and 3.2 times for CD-1, C57Bl/6 and BALB/c mice, respectively. This phenomenon can be explained by the differences in the amount of macrophages in liver and spleen in various mice strains, but also in the dominant immune subtype [220] that influences macrophage activity on recognition and phagocytosis of exogenous substances. Next, the authors studied how chronic pathological conditions caused by a tumor development or acute inflammation influence particle elimination from the bloodstream. In short, the MNPs circulation was prolonged by 5.1 times in the case of melanoma and 2.6 in the case of breast cancer, whereas the inflammation caused 3-fold increase in the time of particle elimination from the bloodstream without blockade. Also, inflammation considerably affected the efficiency of MPS blockade, which changed from 4.2 to 2.8 times.

Liu et al. [217] suggested an approach consisting in targeting the RES and thereby temporarily weakening particle clearance. For this purpose, the authors applied Intralipid, which is a source of parenteral nutrition for patients approved by FDA in 1972. Intralipid 20.0% is composed of 20% soybean oil, 1.2% egg-yolk phospholipids and 2.25% glycerol. Kupffer cells in the liver play an important role in the uptake and metabolism of Intralipid [221]. Superparamagnetic iron oxide nanoparticles “Molday IONC6 Amine” (30 nm in diameter) from BioPAL were used. Intralipid 20.0% was administered by intravenous injection to rats at a 2 g/kg dose. After 1 h, iron oxide particles were injected intravenously at a dose of 4.5 mg Fe/kg of body weight. Pre-treatment with Intralipid resulted in 3-fold increase in the blood half-life of iron oxide nanoparticles: the blood half-life was determined to be 5.1 min, whereas upon Intralipid pre-treatment, the half-life increased to 15.9 min.

Fucoidans belong to the class of safe naturally occurring sulphated polysaccharides. They are ligands of the scavenger receptor class A (SR A) [222] and are also responsible for macrophage uptake of dextran-coated MNPs [223]. The effects of fucoidan on MNPs pharmacokinetics were evaluated using ferucarbotran (carboxy dextran coated nanoparticles), which in its pharmaceutical formulation (Resovist) targets the RES. The results of in vitro studies showed that the pre-treatment with fucoidans resulted in a significant reduction in the clearance rate of ferucarbotran. Also, the results of in vivo studies demonstrated a significant change in the pharmacokinetic behaviour of ferucarbotran in the fucodain-treated mice. For example, the circulation half-life (t_1/2_) of the tested nanoparticles increased 4-fold, from 37.4 to 150 min [224].

Another strategy for extending the circulation time of nanoparticles was put forward by Xu et al. [225]. The authors developed double-PEGylated reduced graphene oxide nanosheets anchored with iron oxide nanoparticles (doubled-PEGylated RGO-INOP) radiolabelled with ^64^Cu for multimodality imaging with enhanced passive tumor targeting capability. The pharmacokinetics of ^64^Cu-RGO-IONP-^1st^PEG and 64Cu-RGO-IONP-^1st^PEG-^2nd^PEG was tested for the distribution half-life (t_1/2α_) and the elimination half-life (t_1/2β_) after an intravenous injection. The short distribution half-life (t_1/2α_) was related to the fast access of MNPs to tissues directly after intravenous injection of the nanoparticles, whereas the long elimination half-life (t_1/2β_) indicated the slow clearance of the nanoparticles from the blood circulation^36^. During the 0–48 h post-injection period, t_1/2α_ of 0.19 h and t_1/2β_ of 18.8 h were calculated in ^64^Cu-RGO-IONP coated with only one type of PEG, whereas after conjugating with ^2nd^PEG, the distribution half-life and elimination half-life were significantly increased to 0.35 and 27.7 h, respectively.

Groult et al. [226] developed a synthesis of oleic-acid (OA)-Fe_3_O_4_ nanoparticles encapsulated into nanomicelles of small phosphatidylcholine (PC) molecule. The analysis of pharmacokinetics of the obtained nanostructures showed extended circulation time of injected PC MNPs for approx. 10 h. This circulation time is remarkably longer than that for MNPs coated with PEG as well as for other structures designed for prolonging the half-time (including polymeric micelles, liposomes or lipoplexes) for which the circulation time in rats was only a few hours [30]. Hence, PC nanomicelles are promising contrast agents for MRI applications. Moreover, small hydrophobic drugs or molecular imaging agents can be easily encapsulated in the nanomicelles together with the (OA)-MNPs leading to potential candidates for a multimodal drug-delivery system.

## Conclusion and perspectives

Magnetic iron oxide nanoparticles are generally considered as biocompatible and safe structures with unique magnetic properties that can be successfully used for biomedical purposes. MNPs are often used as contrast agents in MRI imaging, hyperthermia, the diagnosis and treatment of cancerous tumors, but also countless studies focus on designing magnetic nanoparticles as drug carriers in targeted therapy as well as nanoparticles for the separation of malignant cells.

However, the success of properly designing nanoparticles in the laboratory is determined by their pharmacokinetics, especially in vivo, which, in turn, is determined by many parameters characterizing nanoparticles such as charge and size. The surface charge of MNPs play an important role in the physical stability and influence their interaction with the biological system.

All in all, positively charged MNPs interact strongly with blood components and are cleared relatively quickly from the systemic circulation, contrasting to negatively and neutrally charged MNPs. The most optimal size of MNPs for in vivo use is within the range of 15–100 nm in diameter. Furthermore, the ligands and functional covering molecules often significantly increase the hydrodynamic the MNPs which leads to the macrophage and systemic clearance of designed nanoparticles. Importantly, the toxicity profile of MNPS with functional layers may be changed as a consequence the modification of their biodistribution and clearance. Therefore it is so important to advance in the methods of faster and better determining the pharmacokinetics, biodistribution and toxicology of MNPs. The final pharmacokinetics and blood circulation time depend on the resultant of the above-mentioned and many other. Therefore, despite some general rules, such as the influence of nanoparticle size on the way of their clearance, studies on pharmacokinetics should be performed individually for each designed nanostructure.

In summary, many biological and biochemical processes for various types of magnetic nanoparticles in organisms require systematic research. Biocompatibility, toxicity, targeting efficiency and long-term stability of the functionalized MNPs still remain a challenge. Accurate knowledge of this subject is crucial for introducing nanostructures into clinical applications.

## Data Availability

Not applicable.

## Abbreviations

Ab-RTX: Rituximab antibodies
ACB: AC Biosusceptometry
BSA: Bovine serum albumin
CLSM: Confocal Laser Scanning Microscopy
CMX: Carboxymethyldextran
CTS: Chitosan
DLS: Dynamic Light Scattering
DMSA: Dimercaptosuccinic acid
DOX: Doxorubicin
EDT: Ethylenediaminetriacetete
EPG: O-2-(aminoethyl)polyethylene glycol
EPR: Electron Paramagnetic Resonance
ESR: Electron Spin Resonance
FA: Folic acid
FRS: Ferromagnetic Resonance Spectroscopy
GCPQ: N-palmitoyl-N-monomethyl-N,N-dimethyl-N,N,N-trimethyl-6-O-glycolchitosan
HMNC: Highly magnetic nanocarrier
ICG: Indocyanine green
ICP: Inductively Coupled Plasma
ICP-AES: ICP coupled with Atomic Emission Spectroscopy
ICP-MC: ICP coupled with Mass Spectroscopy
LSEC: Liver sinusoidal endothelial cells
MNPs: (Iron oxide) magnetic nanoparticles
MPI: Magnetic Particle Imaging
MPQ: Magnetic Particle Quantification
MPS: Mononuclear phagocytic system
MSM: Magnetic Susceptibility Measurement
Ner: Neridronate (bisphosphonate anchoring groups)
NHL: Non-Hodgkin lymphoma
PAMAM: Poly(amidoamine) (dendrimer)
PAP: Polyaspartic acid
PAS: Polyacrylic acid
PDI: Polydispersity index
PEG: Poly(ethylene glycol)
PEG/P: Polyethylenglycol α-ω-diphosphate
PEI: Poly(ethyleneimine)
PET: Positron-Emission Tomography
PLGA: Poly(D,L-lactide-co-glycolic) acid
PMAO: Poly(maleic anhydride-alt-1-octadecene)
PMSEA: Poly[2-(methylsulfinyl)ethyl acrylate]
PS: Polystyrenesulfonate
PTT: Photothermal Cancer Therapy
PTX: Paclitaxel
PVP: Polyvinylpyrrolidone
RES: Reticuloendothelial system
SPION: Superparamagnetic iron oxide nanoparticles
SQUID: Superconducting Quantum Interference Device
SR-As: Class A scavenger receptors
TEM: Transmission Electron Microscopy
VEGF: Vascular endothelial growth factor
